# A cancer‐associated *CDKN1B* mutation induces p27 phosphorylation on a novel residue: a new mechanism for tumor suppressor loss‐of‐function

**DOI:** 10.1002/1878-0261.12881

**Published:** 2021-02-06

**Authors:** Debora Bencivenga, Emanuela Stampone, Arianna Aulitto, Annunziata Tramontano, Clementina Barone, Aide Negri, Domenico Roberti, Silverio Perrotta, Fulvio Della Ragione, Adriana Borriello

**Affiliations:** ^1^ Department of Precision Medicine University of Campania “Luigi Vanvitelli” Naples Italy; ^2^ Department of Medicine and Surgery University of Parma Italy; ^3^ Department of Woman, Child and General and Specialized Surgery University of Campania “Luigi Vanvitelli” Naples Italy

**Keywords:** CDK inhibitors, haploinsufficiency, p27 post‐translational modifications, tumor suppressor gene

## Abstract

*CDKN1B* haploinsufficiency promotes the development of several human cancers. The gene encodes p27^Kip1^, a protein playing pivotal roles in the control of growth, differentiation, cytoskeleton dynamics, and cytokinesis. *CDKN1B* haploinsufficiency has been associated with chromosomal or gene aberrations. However, very few data exist on the mechanisms by which *CDKN1B* missense mutations facilitate carcinogenesis. Here, we report a functional study on a cancer‐associated germinal p27^Kip1^ variant, namely glycine9‐>arginine‐p27^Kip1^ (G9R‐p27^Kip1^) identified in a parathyroid adenoma. We unexpectedly found that G9R‐p27^Kip1^ lacks the major tumor suppressor activities of p27^Kip1^ including its antiproliferative and pro‐apoptotic functions. In addition, G9R‐p27^Kip1^ transfection in cell lines induces the formation of more numerous and larger spheres when compared to wild‐type p27^Kip1^‐transfected cells. We demonstrated that the mutation creates a *consensus sequence* for basophilic kinases causing a massive phosphorylation of G9R‐p27^Kip1^ on S12, a residue normally never found modified in p27^Kip1^. The novel S12 phosphorylation appears responsible for the loss of function of G9R‐p27^Kip1^ since S12AG9R‐p27^Kip1^ recovers most of the p27^Kip1^ tumor suppressor activities. In addition, the expression of the phosphomimetic S12D‐p27^Kip1^ recapitulates G9R‐p27^Kip1^ properties. Mechanistically, S12 phosphorylation enhances the nuclear localization of the mutant protein and also reduces its cyclin‐dependent kinase (CDK)2/CDK1 inhibition activity. To our knowledge, this is the first reported case of quantitative phosphorylation of a p27^Kip1^ variant on a physiologically unmodified residue associated with the loss of several tumor suppressor activities. In addition, our findings demonstrate that haploinsufficiency might be due to unpredictable post‐translational modifications due to generation of novel consensus sequences by cancer‐associated missense mutations.

## Graphical abstract

*CDKN1B*, encoding p27^Kip1^, is mutated in human cancers. We demonstrate that c.25G>A (p.G9R) *CDKN1B* mutation, identified in a parathyroid adenoma, generates a new consensus sequence for R‐directed kinases causing p27^Kip1^ phosphorylation on a residue (S12) physiologically unphosphorylated. The unexpected phosphorylation, reducing p27^Kip1^‐dependent cyclin‐dependent kinase inhibition, enhances protein degradation and reduces p27^Kip1^ anticancer activities. Our study unveils an unreported mechanism of tumor suppressor haploinsufficiency.

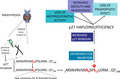

**Abbreviations**
1D/WB: monodimensional western blotting
2D/WB: two‐dimensional western blotting
CDK: cyclin‐dependent kinase
CHX: cycloheximide
G9R‐p27: glycine9‐>arginine‐p27
IUPs: intrinsically unstructured proteins
mAbs: monoclonal antibodies
MEN: multiple endocrine neoplasia
MENX: multiple endocrine neoplasia X
PTMs: post‐translational modifications
rAbs: rabbit antibodies
TSG: tumor suppressor gene
wt‐p27: wild‐type p27

## 1 Introduction

Haploinsufficiency of a tumor suppressor gene (TSG) is an important cause of human cancer. A major example of haploinsufficient TSG is represented by *CDKN1B*, the gene encoding p27^Kip1^ (hereinafter p27). p27 is a well‐known regulator of cell division cycle mostly acting as an inhibitor of cyclin‐dependent kinase 2 (CDK2)‐ and CDK1‐containing complexes and under specific conditions cyclin Ds‐CDK4/6 activator [1, 2, 3, 4, 5, 6, 7, 8]. However, the view of p27 as simply a CDK modulator has been challenged by numerous experiments including studies on genetically modified animals [9, 10].

Several CDK‐independent p27 activities have been identified. Particularly, p27 interacts in the cytosol with RhoA, stathmin, and additional components/regulators of the cytoskeleton, modulating both actin filaments and microtubule dynamics [11, 12, 13, 14, 15, 16, 17].

Cytosolic p27 plays important roles in controlling protein and vesicle trafficking and cell motility [17, 18], and microtubule bundling activity and cytokinesis [19]. Accordingly, it has been considered not only as a protein involved in proper tissue development but also as an oncogenic molecule favoring metastatization and invadopodia formation [11, 12, 13, 14, 15, 16, 20].

The protein has also been consistently demonstrated to regulate apoptosis and autophagy, transcription, the DNA damage response, and prereplicative and replicative complex formation [21, 22, 23, 24, 25]. Recently, in nontumoral cardiac cells, p27 has been localized in mitochondria where it improves the organelle functions [26]. In brief, the increasing number of p27 interactors has enlarged vertiginously the spectrum of CDK‐independent p27 roles [18, 29].

Structurally, p27 is the prototype of intrinsically unstructured proteins (IUPs) [30, 31]. The intrinsic scarce degree of defined tertiary structure gives to IUPs the necessary conformational flexibility to exert a multiplicity of functions. Moreover, IUPs undergo to multiple post‐translational modifications (PTMs) that address these proteins toward specific structural organizations affecting their levels and functions [30, 31]. Accordingly, several p27‐phosphorylated residues have been identified, although only in few cases their functions have been definitely unraveled. T187 phosphorylation, mostly catalyzed by active CDK complexes, induces p27 binding to Skp2, the substrate‐recognition component of E3 SCF ligase, allowing nuclear protein ubiquitination and removal in S/G2 phases [32, 33]. T157 and/or T198 p27 phosphorylations have been associated with protein sequestration in the cytosol and cytoskeletal remodeling [16, 28, 34]. Very recently, these PTMs have been correlated with the regulation of c‐Jun activity as transcriptional factor [25]. T198 phosphorylation has also been associated with protein stability and with the decision between distinct cellular responses to stress conditions [28, 35]. Y88/89 modifications, attributed to nonreceptor tyrosine kinases, switch p27 from being an inhibitor to become a CDK2 substrate, enhancing T187 phosphorylation and its ubiquitin‐dependent degradation [36]. The most abundant p27 PTM is S10 phosphorylation, which has been reported to stabilize the protein in the nuclear compartment and/or to induce p27 exit from the nucleus [37, 38, 39, 40].

In mice, *Cdkn1b* knockout leads to increased body size, hyperplasia of different organs, and development of pituitary adenomas [41]. Moreover, *Cdkn1b^+/−^* mice show a major susceptibility to chemical carcinogens‐ or irradiation‐induced tumors [42]. In rats, a germline *Cdkn1b* homozygous inactivation has been identified as causative of a form of multiple endocrine neoplasia, called multiple endocrine neoplasia X (MENX). More recently, Pellegata *et al*. reported that rat hemizygotes for *Cdkn1b* mutation develop the same spectrum of MENX, although with a slower progression [43]. These data allowed the proposal that *Cdkn1b* is a haploinsufficient TSG. The suggestion is confirmed by data from human cancers. For many years, p27 decrease has been described in numerous human tumors, such as colon, breast, prostate, and ovarian carcinomas, associated with a major aggressiveness. The protein reduction was reported to occur mainly through altered PTMs inducing increased p27 degradation or cytoplasmic mislocalization [44].

However, due to NGS and genome‐wide association analysis development, *CDKN1B* has been now found mutated in several cancers. Particularly, *CDKN1B* represents one of the 18 most significantly mutated genes in luminal breast cancer, a subtype accounting for more than 60% of all breast cancers [45]. Also, p27 downregulation predicts resistance to radiotherapy and anti‐HER2 therapies [46]. *CDKN1B* was also found remarkably mutated in prostate cancers [47], and it has been identified as the second most frequently altered gene, after *BRAF*, in hairy cell leukemia [48]. *CDKN1B* is also frequently heterozygously inactivated in small intestine neuroendocrine tumors [49, 50], in sporadic parathyroid adenomas [51] and germline inactivated in MEN4, a newly defined subtype of MEN, human autosomal dominant disorders characterized by the occurrence of spread endocrine tumors [52, 53, 54]. These observations allow to define *CDKN1B* as the archetype of haploinsufficient TSGs. Among the *CDKN1B* mutations found in cancers, many are nonsense or small deletions/insertions causing the synthesis of truncated p27 variants, with deleterious effects on protein levels and functions [29, 55]. *CDKN1B* missense mutations have also been reported, but only few pieces of information on their effects on the protein structure/function are available [53]. Thus, the mechanistic interplay between *CDKN1B* missense variants and p27 activity is very far to be clarified.

In this study, we report a detailed functional characterization of a cancer‐associated *CDKN1B* missense mutation and evidenced an unexpected and undescribed mechanism by which the genetic change might result in a haploinsufficient phenotype. Our findings allow the proposal of an unprecedented general mechanism for the development of haploinsufficiency in which a cancer‐associated massive phosphorylation of a physiologically unmodified residue results in the loss of the tumor suppressor functions.

## 2 Materials and methods

### 2.1 Materials

MG132 and autocamtide‐2‐related inhibitory peptide (AIP) were supplied by Sigma Chemical Company (St. Louis, MO, USA). λ protein phosphatase was obtained from Santa Cruz Biotechnologies (Santa Cruz, CA, USA). The Screen‐Well Kinase Inhibitor Library, E‐64, and human recombinant CDK2/CycE‐GST and CDK1/Cyclin A2 were furnished by Enzo Life Sciences Inc. (Farmingdale, NY, USA). Human wild‐type (wt)‐p27 and S10A‐p27 coding sequences, cloned into the pcDNA3.0 plasmid, were gently given by M. Pagano (Dpt. Biochemistry and Molecular Pharmacology, Perlmutter Cancer Center, New York University, New York, NY, USA). QuikChange II Site‐Directed Mutagenesis Kit was from Agilent Technologies (Santa Clara, CA, USA). TNT Quick Coupled Transcription/Translation System and ADP‐Glo Kinase Assay kit were purchased from Promega (Madison, WI, USA). Human recombinant Rb/p107 was from Merck KGaA (Darmstadt, Germany).

### 2.2 Antibodies, mono‐ and two‐dimensional western blotting, and immunoprecipitation

Monoclonal antibodies (mAb) to p27 were from BD Transduction Laboratories (AB_397637, Franklin Lakes, NJ, USA). Rabbit Ab (rAb) to p27(C19) (AB_632129), pS10‐p27 (AB_2260344), pT187‐p27 (AB_670358), Cdc2 p34 (CDK1, AB_631207), CDK2 (AB_631215) and HDAC1 (AB_2279709), PKM2 (AB_10844484), Cdc2 p34 (AB_627224), CDK2 (AB_627238), and affinity purified normal rabbit IgGs and mouse IgGs were from Santa Cruz Biotechnology. rAb against Actin (AB_476693) was from Merck KGaA. Phospho‐Rb (Ser807/811) antibodies (D20B12; Cell Signaling Technology) and mAb to Rb total (Rb1 1F8, ab24; Abcam, MA, USA) were also employed.

Immunoprecipitation, mono‐ and two‐dimensional western blotting (1D/WB and 2D/WB) were performed as reported [40]. The lambda protein phosphatase treatments were done on extracts, recombinant mutant proteins, or immunoprecipitated proteins, as reported previously [40]. Densitometry analysis of 1D and 2D western blottings was done using imagej software or, alternatively, TotalLab CLIQS gel image analysis Software (TotalLab Ltd 2019, Newcastle‐Upon‐Tyne, UK).

### 2.3 Cell lines, treatments, and cellular fractionation

LN‐229 was obtained from ATCC (Manassas, VA, USA). Other cell lines were available in our laboratory and were authenticated using short‐tandem repeat DNA profiling. Mouse embryonal fibroblasts (MEFs) and *Cdk2* ^–/–^, *Cdk4* ^–/–^, and *Cdk2* ^–/–^ *Cdk4* ^–/–^ knockout MEFs were gently given by M. Malumbres and M. Barbacid (Cell Division and Cancer Group, Spanish National Cancer Research Centre, Madrid, Spain). PC‐3 cells were maintained in Dulbecco's Modified Eagle's medium (DMEM)/F‐12 Ham supplemented with 10% FBS, 1% penicillin/streptomycin, and 1% glutamine. Growth conditions of LN‐229, HeLa, SH‐SY5Y, and K562 cell lines were as reported [56]. Whole‐cell extracts were obtained as previously described [57]. Nuclear and cytosol extracts were prepared using CelLytic NuCLEAR Extraction Kit (Merck KGaA) following manufacturer's instructions or as in Ref [58] and tested for cross‐contamination as reported [40].

### 2.4 Plasmid preparation and transfection

Point mutations were introduced into a pcDNA3.0 plasmid containing human wt‐p27 coding sequence as reported [40]. Mutagenesis was confirmed by sequence analysis. The oligonucleotide sequences employed for mutagenesis are available on request. Cell transfection was performed using Lipofectamine 3000 (Thermo Fisher Scientific, Inc, Waltham, MA, USA), following manufacturer's instructions, except for K562 cell line, for which electroporation was performed using Gene Pulser Xcell electroporation system (Bio‐Rad, Hercules, CA, USA) as in Ref. [59].

### 2.5 Apoptosis detection, wound‐healing, and Transwell migration assays

Apoptosis induction was evaluated as follows. 1.5 × 10^5^ PC‐3 cells were seeded in 6‐well plates, cultured for 24 h, transfected with the indicated plasmids or empty vector, and collected at the time specifically reported in figure legends using Accutase (Thermo Fisher Scientific, Inc). Then, the transfected cells were resuspended at 1 × 10^6^ cells/mL prior to staining with Alexa Fluor 488 Annexin V/Dead Cell Apoptosis Kit (Thermo Fisher Scientific, Inc) as suggested by the manufacturer's instructions. Apoptotic analysis was performed on a FACSCalibur (Becton Dickinson, Franklin Lakes, NJ, USA), using the cell quest Software (Becton Dickinson) and calculated on at least 20 000 events. To evaluate effects of wt‐p27, glycine9‐>arginine‐p27 (G9R‐p27), and its derivatives on staurosporine‐induced apoptosis, PC‐3 cells, after 48 h of transfection with the selected plasmids, were treated with 1 µm staurosporine for 18 h and processed as indicated above. Cell apoptosis was calculated on 50 000 events. The Transwell migration assay was carried out using Boyden chambers, consisting of 8‐µm polycarbonate membrane Transwell inserts (12 wells) (Thermo Fisher Scientific Inc.) using LN‐229 cells. The cells were transfected with indicated constructs or empty vector; after 48 h, they were dissociated into single cells using Accutase (Thermo Fisher Scientific, Inc.) and seeded on the top of the Transwell inserts at a density of 20 000 per filter. Cells were incubated with 0.1% FBS containing DMEM, while 10% FBS containing medium was added outside the Transwells as a chemotactic agent. At 24 h postplating, noninvaded cells on the top of the membranes were removed using a cotton swab and migrated cells were fixed with ice‐cold 100% methanol, stained with crystal violet, and photographed at 20× magnification using an inverted phase contrast microscope. The wound‐healing assays were performed in LN‐229 cells as described [60].

### 2.6 Immunofluorescence microscopy

Cell lines were grown in 4‐well tissue culture chambers (Sarstedt, Nümbrecht, Germany) and transfected for 24 h with the indicated constructs. After fixation with 4% (p/v) paraformaldehyde at room temperature for 15 min, the cells were permeabilized with PBS/0.1% Triton X‐100 for 10 min. After incubation with 5% horse serum/0.05% Triton X‐100 (blocking buffer) for 1 h, the glasses were incubated with anti‐p27 mAb overnight at 4 °C. The immunostaining was performed by incubation with Alexa Fluor 488‐conjugated goat anti‐mouse IgG (Abcam) at room temperature for 1 h under mild agitation in the dark. The slides were then stained with Phalloidin CruzFluo 555 Conjugate (Santa Cruz Biotechnology) and Hoechst 33342 Trihydrochloride Trihydrate (Thermo Fisher Scientific) for 20 and 10 min, respectively. Fluorescent images were obtained using a Carl Zeiss (Oberkochen, Germany) LSM 700 confocal laser scanning microscope through a 63X/1.4 PlanApo oil, and almost five images from each slide were selected randomly for imaging.

### 2.7 Phos‐tag SDS/PAGE

To investigate the phosphorylation status of transfected p27 mutants, total extracts from PC‐3 cells harvested after 24 h of transfection were processed for Phos‐Tag SDS/PAGE using Phos‐tag acrylamide (Wako Chemicals USA, Inc., Richmond, VA, USA), as indicated by the manufacturer. The phosphorylation of each p27 mutant was evaluated by means of SDS/PAGE using 100 μm Phos‐tag Acrylamide, 8% polyacrylamide gel followed by immunoblotting using anti‐p27 mAb.

### 2.8 Spheroid formation

To analyze the effect of G9R‐p27 expression on the growth without adhesion and tumor invasion capacity of the glioblastoma multiforme cell line LN‐229, we use a 3D spheroid‐based tumor invasion assay starting from three different cell sources, that is, vehicle‐treated cells, WT‐ and G9R‐p27 overexpressing cells, plated at higher confluence (70–80%). After 24 h of transfection, cells were detached using accutase and centrifuged (200 ***g***, 5 min). A control of transfection for each sample was harvested, lysed, and analyzed by western blotting using anti‐p27 mAb to verify comparable level of protein expression. For spheroid formation, cells were resuspended in serum‐free medium, dissociated into single‐cell suspensions using trituration, prior to subculturing in cold 3D Corning® Matrigel® Growth Factor Reduced Basement Membrane Matrix, diluted with each cell suspension just to 3 mg·mL^−1^ ECM proteins and 2.0 × 10^3^cells/well (in a 12 well‐plate), in a final volume of 1 mL per well. All the experiments were carried out in triplicates. Tumorspheres were observed under a light microscope every day for 1 week and photographed at 4th, 5th, and 6th day, choosing randomly five fields/treatment. The experiments were carried also on PC‐3 cells, following exactly the above‐reported procedures.

Final images were processed using imagej software to measure colony diameters comparing to the scale bar, enabling accurate analysis of the growth without adhesion and the invasive capabilities of 3D spheres over time. Magnified images evidence individual cell migration.

### 2.9 Kinase inhibitor screening

The Screen‐Well Kinase Inhibitor Library was used to characterize kinases involved in G9R‐p27 or S10/G9R‐p27 phosphorylation. Forty‐seven out of 80 drugs furnished by this library and AIP were used to treat PC‐3 cells starting from 2 h before 24 h of transfection. Further details are in Table 1. The cells treated with drugs or DMSO were collected, and total cell extracts were analyzed through 2D/WB.

**Table 1:** Compounds tested as inhibitors of G9R‐p27 phosphorylation of serine 12. The compounds reported in the Table were added at the showed concentrations to PC‐3 cultures 2 h before pcDNA3.0 G9R‐p27 or S10A/G9R‐p27 transfection. After 24 h of transfection, PC‐3 cellular extracts were prepared and analyzed by 2D/WB. The bidimensional patterns were analyzed by imagej software to estimate the percentage of inhibition. The values are the mean ± SD of, at least, three separate experiments. S10A/G9R‐p27 transfection was used to evaluate only S12 G9R‐p27 phosphorylation. ND, not done.

Compounds	Target	Conc (µm)	Inhibitory effect on G9R‐p27 phosphorylation (%)	Inhibitory effect on S10A/G9R‐p27 Phosphorylation (%)
PD‐98059	MEK	10	0	20 ± 5
U‐0126	MEK	10	0	0
SB‐203580	P38 MAP Kinase	10	40 ± 5	ND
H‐7‐2HCl	PKA, PKG, MLCK and PKC	5	0	0
H‐9‐2HCl	PKA, PKG, MLCK and PKC	5	0	0
Staurosporine	Pan‐specific	0.02	0	0
Tyrphostin 46	EGFRK, PDGFRK	50	0	0
PKC‐412	PKC Inhibitor	5	0	0
AG‐490	JAK‐2	5	0	0
LY 294002	PI 3‐K	10	0	0
Wortmannin	PI 3‐K	5	0	0
GF 109203X	PKC	5	0	0
Hypercin	PKC	10	0	0
Ro 31‐8229 mesylate	PKC	5	ND	40 ± 10
D‐erythro‐sphingosine	PKC and CaMK	5	0	0
H‐89‐2HCl	PKA	1	0	0
2‐Hydroxy‐5‐(2,5‐dihydroxybenzylamino) benzoic acid	PKA	5	ND	0
KN‐62	EGFRK, CaMK II	1	60 ± 10	70 ± 10
KN‐93	CaMK II	1	ND	40 ± 10
ML‐9‐HCl	MLCK	10	ND	0
N9‐Isopropyl‐olomucine	CDK	2	ND	0
Olomucine	CDK	100	ND	0
Roscovitine	CDK	20	20 ± 5	0
5‐Iodotubercidin	ERK2, Adenosine Kinase, CKI, CK2II	1	0	ND
LFM‐A13	BTK	10	0	0
SB‐202190	P38 MAPK	2	ND	0
ZM336372	cRAF	5	0	0
SU413	Flk	1	0	0
GW5074	cRaf	1	0	0
Palmytoil‐DL‐carmitine	PKC	10	ND	70 ± 10
Rottlerin	PKC delta	10	ND	0
Genistein	Tyrosine Kinase	1	ND	0
Quercetin‐2H2O	PI 3‐K	5	0	0
Bay 11‐7082	IKK pathway	10	ND	0
5.6‐Dichloro‐1beta‐D‐ ribofuranosylbenzimidazole	CKII	10	0	0
2,2,3,3’,4,4’‐Hexahydroxy‐1,1’‐biphenyl,‐6,6’.dimethanol dimethyl ether	PKC alpha, PKC delta	1	ND	0
SP 600125	JNK	5	0	0
Indirubidin	GSK‐3 beta, CDK5	10	ND	0
Indirubidin‐3’‐monoximine	GSK‐3 beta	10	0	ND
Y‐27632‐2HCl	ROCK		0	0
Kenpaullone	GSK‐3 beta	10	0	0
Terreic acid	BTK		0	0
Triciribine	Akt signaling pathway	10	0	0
BML‐257	Akt	10	ND	0
SC‐514	Ikk2	1	ND	0
BML‐259	CDK5/p25	0.5	0	0
Apigenin	CK‐II	20	0	0
Rapamycin	mTor	1	0	0
AIP	CamK II	1	ND	30 ± 10 70 ± 10^a^

^a^ The AIP inhibitory activity was also estimated by adding the compound to IVTT assay of S10A/G9R‐p27.

### 2.10 *In vitro* kinase assays

All the kinase assays were performed using p27 mutants obtained through overexpression in PC3 cell line or through IVTT. Either total cell extract (obtained by freezing‐thawing) or IVTT mixtures were treated at 90 °C for 2 min for p27 partial purification and centrifuged at 10 000 ***g***. The supernatants containing thermostable p27 were filtered and diluted several times in Amicon Ultra Centrifugal Units (10K cutoff; Merck Millipore, Ltd) for removing small proteins and molecules (including nucleotides). To evaluate Thr187 phosphorylation, 100 ng of p27 (wt or mutants) was incubated with recombinant Cyclin E/CDK2‐GST in kinase buffer (50 mm Tris/HCl pH 7.5, 10 mm MgCl_2_, and 1 mm DTT, protease and phosphatase inhibitor cocktails) with 150 µm ATP at 30 °C for 30 min. The reaction was stopped with SDS/PAGE loading buffer. The samples were subjected to 12% SDS/PAGE and analyzed by immunoblotting using anti‐(pT187)p27 antibody. Alternatively, the kinase assay was blocked by the addition of 6 m urea and then analyzed by 2D SDS/PAGE/immunoblotting using monoclonal anti‐p27 antibody. The T187 phosphorylation was quantified comparing the (phospho)isoforms ratio to the respect of the control without enzyme.

To test wt‐p27 and G9R‐p27 inhibitory effects on CDK2 activity, two distinct assays with different substrates (p27 or Rb) were performed. Particularly, 50 ng of recombinant proteins (S10A/T187A‐p27 or S10A/T187A/G9R‐p27) was incubated with recombinant cyclin E/CDK2‐GST in a binding buffer (50 mm Tris/HCl, pH 7.5, 3 mm MgCl_2_, 3 mm MnCl_2_, 3 µm sodium orthovanadate, 1 mm DTT). After 10 min, 150 µm ATP and dephosphorylated recombinant wild‐type p27 (wt‐p27) were added as CDK2 substrate; then, the mixture was incubated for 30 min at 30 °C. Equal volumes of the reaction mixtures were then analyzed by immunoblotting employing anti‐pT187‐p27 antibodies. Alternatively, the kinase assay was blocked by addition of 6 m urea and then analyzed by 2D SDS/PAGE/immunoblotting using monoclonal anti‐p27 antibody.

The inhibition of p27 proteins on CycE/CDK2‐GST enzymatic activity was also evaluated using recombinant human full‐length Rb as substrate and phospho‐Rb (Ser807/811) antibodies as revealing tool. WT‐ or G9R‐p27 proteins (partially purified from transfected cells, as described above) were added to CycE/CDK2 complexes incubated in a kinase buffer (50 mm Tris/HCl pH 7.5, 20 mm MgCl2, 150 μm ATP, protease, and phosphatase inhibitors). A control was performed using equally treated extract from cells transfected with empty vector. Dephosphorylated human retinoblastoma protein (p107) was added as substrate. The mixtures were left to proceed under mild agitation for 1 h at 30 °C. Equal volumes of the reaction mixtures were then analyzed by immunoblotting employing anti‐phospho‐Rb(Ser807/811) antibodies. Total Rb, CDK2, and p27 levels were also evaluated by western blotting in the kinase assay samples.

CycA/CDK1‐GST enzymatic activity was evaluated using ADP‐Glo kinase assay system (Promega Corporation). CycA/CDK1 complex was first incubated with recombinant p27 proteins in kinase buffer without ATP for 20 min at 30 °C, and then, 100 ng of Histone 1 and 150 µm ATP were added and the assay was then performed following manufacturer's instructions.

### 2.11 Protein degradation analysis

K562 cells were transfected with wt‐, G9R‐p27, or empty vectors for 16 h; afterward, 3.6 µm cycloheximide (CHX) was added for 6 h. During the last 2 h of CHX incubation, proteasome (MG132, 1 µm) or lysosome protease (E‐64, 10 µm) inhibitors were added to the cell cultures. 10 µg of total extracts was analyzed by SDS/PAGE/WB using anti‐p27 or anti‐actin.

### 2.12 Statistical analysis

Experimental data were expressed as mean ± SD. graphpad prism 6 (GraphPad Software, La Jolla, CA, USA) was used for statistical analysis. Comparisons among samples were performed using sample *t*‐test or ANOVA. *P*‐value < 0.05 was considered significantly different.

## 3 Results

### 3.1 G9R‐p27 lacks p27 tumor suppressor activities

G9R‐p27 was identified as a heterozygous germline mutation (c.25G>A pG9R) in a parathyroid tumor [51]. Initially, we compared the main tumor suppressor activities of G9R‐p27 to those of p27. HEK‐293 cells and MEFs were transfected with plasmids encoding wt‐p27 and G9R‐p27. The two embryonic cell models are particularly suitable for evaluating effects of exogenously expressed proteins, due to their high transfection efficiency. Preliminary, we evaluated by FACS analysis the percentage of cells (HEK‐293) expressing both exogenous wild‐type (wt) and mutated p27. The data obtained indicated that, at 48 h upon transfection, at least 50% of cells express the proteins (Fig. S1).

**Fig. 1:**
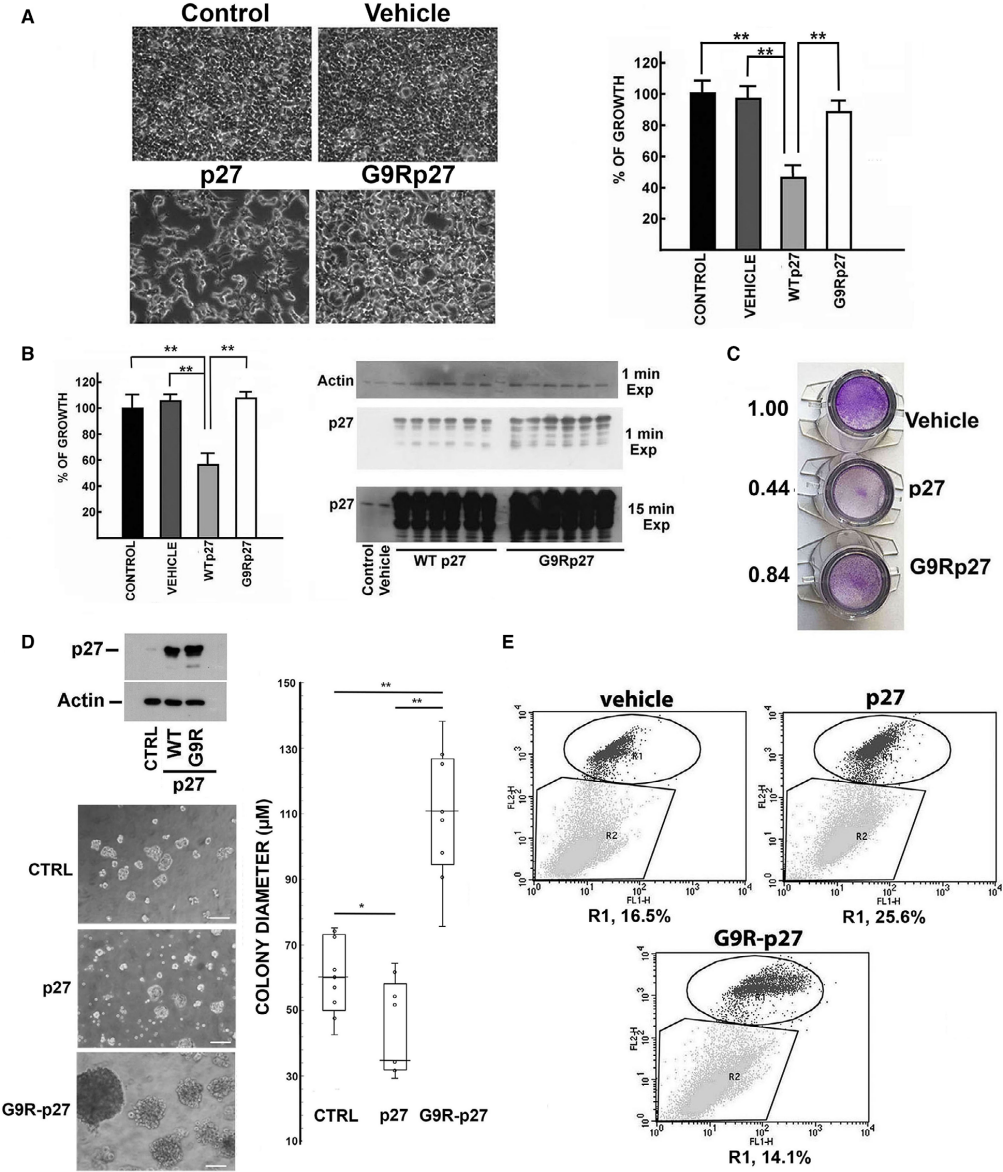
Phenotypical Effects of G9R‐p27 exogenous expression. (A) HEK‐293 cells were transfected with different pcDNA3.0 plasmids, namely empty plasmid (defined as ‘Vehicle’) and plasmids encoding p27, and G9R‐p27. Untransfected HEK‐293 cells are showed as ‘Control’. After 72 h of transfection, microscopy images were taken (10×) (on the left). On the right, the results of cell counting are reported. The data showed are the mean of three independent experiments, and the standard deviation is reported. Data were analyzed by Student's *t*‐test. **P < 0.01. (B) MEF cells were transfected with different pcDNA3.0 plasmids, namely empty plasmid and plasmids encoding p27 (WTp27), and G9R‐p27. On the left. Cells transfected with the empty plasmid are defined as ‘vehicle’. Untransfected MEF cells are showed as ‘control’. Three independent transfections were performed on two distinct wells. After 72 h of transfection, the cell number of each well was counted. The data showed in the graph on the left are the mean of the six counts, and the standard deviation is reported. Data were analyzed by Student's *t*‐test. **P < 0.01. On the right. MEF cells from the experiments described in Panel B were used to prepare cell extracts. These extracts were analyzed by WB for evaluating p27 or G9R‐p27 content in the transfected cells. In particular, we analyzed one sample from untransfected cells (control) and one from MEF cells transfected with the empty plasmid (vehicle). Conversely, all the extracts of transfected cells were analyzed. In addition, the films were exposed for two different times to the filter as indicated to show p27 content in control and vehicle cells. Actin was evaluated as loading control. (C) Transwell migration assay was performed as described in Materials and methods. Briefly, LN‐229 cells were transfected with the plasmids reported in the image. Vehicle represents cells transfected with the empty vector. After 48‐h transfection, cells were collected and an equal number of cells (20 000 cells) were seeded on a transwell insert. 24 h later, the insert was removed and treated as reported under Materials and methods. Crystal violet staining was used to evidentiate the cells that have migrated through the membrane. On the left of the images, the results of a densitometric analysis of the staining intensities of the total membrane areas are shown. (D) 3D spheroid‐based tumor invasion assay was performed as reported in ‘Materials and methods’ Section, starting from LN‐229 glioblastoma cell line transfected the day before with empty vector (CTRL) or plasmids encoding p27, and G9R‐p27. On the left: top) comparable levels of transfection were evaluated by WB using anti‐p27 mAb. Actin was used as loading control; bottom) light microscope images of tumorspheres formed by the different cell populations at the 6th day of culture. On the right, the diameter of the colonies obtained in the different conditions was measured using the scale bar (50 µm) as reference. The results shown are the mean of 9 determinations obtained on three independent experiments, and standard deviation is showed. Data were analyzed by Student's *t*‐test. *P < 0.05; **P < 0.01. (E) PC‐3 cells were transfected for 5 days with pcDNA3.0 empty vector (Vehicle), and pcDNA3.0 encoding p27 or G9R‐p27. Then, the cells were collected and processed with Alexa Fluor 488 Annexin V/Dead Cell Apoptosis Kit according to manufacturer's indications. Cell apoptosis was detected by flow cytometry using a FACSCalibur and calculated from 20 000 events. Further details are reported under Materials and methods. R1 includes apoptotic cell populations expressed as percentage of total analyzed events.

As shown in Fig. 1A (cell images and histogram), wt‐p27 reduced significantly HEK‐293 proliferation while, unexpectedly, G9R‐p27 scarcely affected cell growth. Similarly, in MEFs (Fig. 1B, left) the expression of the wt protein inhibited proliferation to an extent of 53 ± 13% after 48‐h transfection, while the G9R variant was unable to affect cell growth. As control for this experiment, the levels of wt‐p27 and G9R‐p27 were determined by immunoblotting (Fig. 1B, right). The cell content of G9R‐p27 was even higher than the wild‐type counterpart, although unable to inhibit the proliferation activity (Fig. 1B).

Transwell migration assays were then performed to compare the activity of wt‐p27 and G9R‐p27 on cell movement and invasiveness. Human glioblastoma LN‐229 cells were used for this purpose due to their reported high migration properties. The transfected cells migrated through the membranes were stained using crystal violet. While wt‐p27 strongly downregulated cell migration, G9R‐p27 scarcely inhibited cell movement (Fig. 1C). LN‐229 cells have also been reported capable to grow as spheres when cultured in plates coated with Matrigel and in serum‐free medium [61]. Thus, we evaluated the behavior of LN‐229 exogenously expressing wt‐p27 or G9R‐p27 in 3D‐spheroid‐based tumor invasion assay. We used cells transfected with empty vector as control. The transfection efficiency and comparable levels of the expressed proteins were confirmed by immunoblotting, as reported in Materials and methods (Fig. 1D, top). As shown in microscopy images and in the quantitative evaluation of the spheroid diameters reported in Fig. 1D, while p27 expression reduces the dimension of glioblastoma spheres, G9R‐p27 tumoroids increase in size statistically and significantly compared with wt‐p27‐ or vehicle‐derived colonies after 6 days from seeding. The experiment was performed three times, and images were taken at different days after seeding in Matrigel (at 4, 5, and 6 days) and reported in Fig. S2. Calculation was obtained from nine different sphere diameter determinations. It is to note that images obtained after 6 days show the presence of cells that appear detaching from spheres (see the inset in Fig. S2) disclosing the ability to invade the protein matrix. Given the impressive behavior, especially when comparing the G9R‐expressing cells to the control (cells transfected with the empty vector), the experiment was repeated in a different cellular model, that is, PC‐3 cells. The results, reported in Fig. S3, confirmed the wt‐p27 inhibitory effects on 3D‐culture development. In this setting, G9R‐p27 disclosed an invasive capacity similar to that of control cells. The differences observed with respect of the data reported in Fig. 1D are probably due to the different cell contexts.

It has been reported that, in some models, p27 expression increases apoptosis either *per se* or potentiating pro‐apoptotic agents [27]. Figure 1E shows that in PC‐3 cells, after 5 days upon transfection, G9R‐p27 variant completely lacks the pro‐apoptotic activities showed by wt‐p27. Moreover, as reported in Fig S4, while p27 works in epistasis with staurosporine enhancing its pro‐apoptotic capacities, G9R‐p27 is unable to increase the staurosporine‐induced programmed cell death.

In summary, the change in glycine 9 into arginine results in a clear loss of important tumor suppressor activities of p27.

### 3.2 G9R substitution induces a quantitative novel p27 phosphorylation

Post‐translational modifications strongly affect the properties of p27, being the protein an IUP. Thus, we asked whether G9R mutation might affect p27 postsynthetic modifications.

**Fig. 2:**
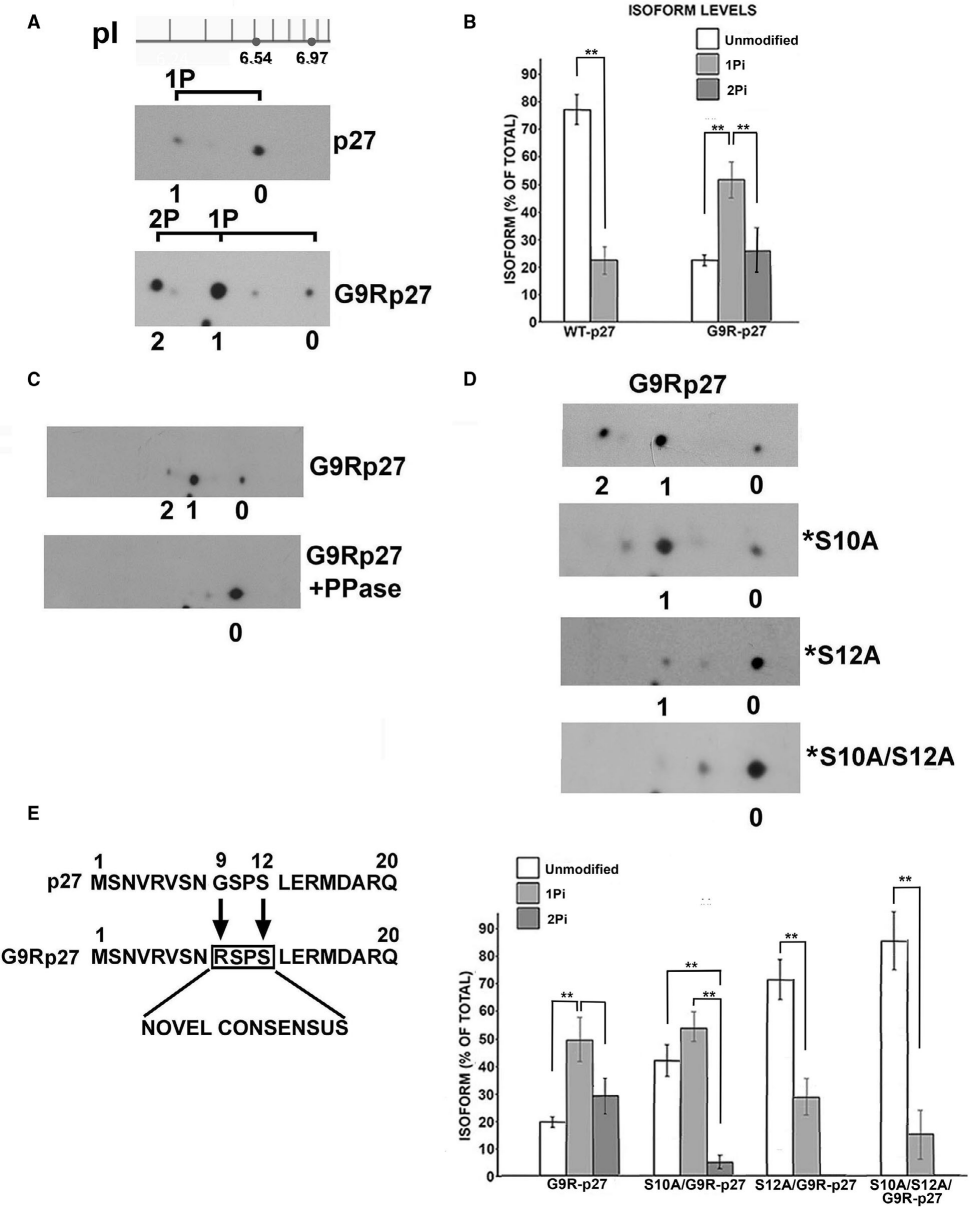
Biochemical characterization of G9R‐p27 isoform pattern. (A) Cell extracts from PC‐3 cells transfected with plasmids encoding p27 or G9R‐p27 were analyzed by 2D/WB using anti‐p27 mAb. A pH scale is shown above the filter images. As reported in previous studies (31), unmodified p27 (pI = 6.54) and monophosphorylated p27 (1P) correspond to signals 0 and 1, respectively. On the basis of theoretically determined isoelectric points, unmodified G9R‐p27 (pI = 6.97), monophosphorylated (1Pi)‐G9R‐p27, and diphosphorylated (2Pi)‐G9R‐p27 correspond to signals 0, 1, and 2, respectively. Further details are reported under Materials and methods Section. (B) The histogram reports the percentage of the relative signal intensity of each spot in respect of the total for the 2D analysis of wt‐p27 and G9R‐p27 proteins. The data shown are the results of three independent experiments. The intensities of the specific signals were evaluated using TotalLab CLIQS gel image analysis Software. Bars represent standard deviation. Data were analyzed by Student's *t*‐test. **P < 0.01. (C) Two aliquots of PC‐3 cell extracts transfected with plasmid encoding G9R‐p27 were prepared. One aliquot, prepared in a buffer without inhibitors of phosphatase, was treated with protein phosphatase lambda (PPase). The other aliquot was in a buffer including phosphatase inhibitors. Both samples were analyzed by 2D/WB as reported in panel A. Further details are under Materials and methods Section. Signals 0, 1, and 2 correspond to unmodified, 1Pi‐protein, and 2Pi‐protein, respectively. (D) Upper Panel. PC‐3 cells were transfected with four different pcDNA3.0 plasmids encoding G9R‐p27, and S10A/G9R‐p27 (*S10A), S12A/G9R‐p27 (*S12A), and S10A/S12A/G9R‐p27 (*S10A/S12A). After 24‐h transfection, cell extracts were prepared and equal amounts of proteins were analyzed by 2D/WB employing anti‐p27 mAb. Further details are under Materials and methods Section. Signals 0, 1, and 2 correspond to unmodified, 1Pi‐protein and 2Pi‐protein, respectively. Bottom Panel. The histogram reports the intensity percentage of each signal (unmodified, 1Pi‐ and 2Pi‐isoforms) relative to the total for G9R‐p27 and its derivative mutant proteins. The intensity of the specific signals was evaluated using TotalLab CLIQS gel image analysis Software. The data shown are the results of three independent experiments. Bars represent standard deviation. Data were analyzed by Student's *t*‐test. **P < 0.01. (E) Sequence alignment between N‐terminal sequence of p27 and G9R‐p27. The G9R substitution creates in the mutant protein a strong *consensus* at +3 amino acids (in the box, on S12) for arginine‐directed kinases.

The coding sequences of wt‐p27 and G9R‐p27 were transfected in PC‐3 cells. Extracts of the transfected cells were then analyzed by bidimensional electrophoresis followed by immunoblotting (2D/WB). While wt‐p27 showed the previously described p27 2D‐pattern [40, 62, 63], characterized by a strong signal corresponding to the unmodified protein (signal 0) and another spot assigned to a monophosphorylated form (signal 1P), G9R‐p27 mutant showed an unusual 2D/WB configuration (Fig. 2A). Particularly, as expected, G9R‐p27 unmodified isoform (signal 0) focalizes at a higher pH compared to that of wt‐p27 unmodified form (6.97 versus 6.54). The pI change is due to the presence of the positively charged arginine in the place of glycine‐9. More intriguingly, a dramatic accumulation of the putative (on the basis of the specific pI) monophospho‐isoform(s) (1P‐isoforms) spot was observed (signal 1), along with a strong signal probably corresponding to 2P‐isoform(s) (signal 2). The relative intensities of the spots corresponding to the different isoforms were determined as in Materials and methods and are shown in Fig. 2B. The histogram in Fig. 2B has been obtained from 3 independent experiments, and the statistical significance is also shown. To identify definitely signals 1 and 2 as G9R‐p27 phosphoderivatives, we treated cell extracts with protein phosphatase lambda. As shown in Fig. 2C, a complete disappearance of signals 1 and 2 was observed, concomitantly with the increase of signal 0. It is worth noting that a further spot occurs between the unmodified and monophosphorylated isoforms. A similar form (together with its putative monophosphorylated derivative between signals 1 and 2) has been observed by us and others in the 2D/WB analysis of wt‐p27 and corresponds to an uncharacterized PTM (distinct from phosphorylation) of p27 [62, 63]. For sake of clarity, other spots are observable in this and other p27 2D patterns, occurring at a lower molecular weight: they have been associated by us to the use of a second AUG codon occurring in the coding sequence of 27.

Since the large steric hindrance of R9 side‐chain might affect (negatively or positively) the phosphorylation of the contiguous S10 residue, we investigated the bidimensional pattern of a G9R‐p27 mutant in which serine 10 was substituted with alanine. Thus, an expression vector for the mutant S10A/G9R‐p27 was prepared and transfected in PC‐3 cells; then, the cell extract was analyzed by 2D/WB. As clearly shown in Fig. 2D, signal 2 (2P‐protein) almost completely disappeared. We concluded that G9R‐p27 is phosphorylated on S10 and that the 2P‐isoform of G9R‐p27 contains pS10, in addition to a further phosphorylation on a different residue(s). It is interesting to report that anti‐pS10 p27 antibodies were unable to recognize their antigen on G9R protein, probably because of steric hindrance. To identify the second residue(s) subjected to detectable phosphorylation, we prepared and transfected S10A/G9R‐p27‐expressing plasmids further mutated on the major known phosphorylatable p27 residues, namely T187A (S10A/T187A/G9R‐p27), T198V (S10A/T198V/G9R‐p27), T157A (S10A/T157A/G9R‐p27), and the three tyrosines, Y74,88,89F [S10/(Y74,88,89F)/G9R‐p27]. The data obtained, reported in Fig. S5A (on the left), allow to rule them out as major sites of G9R phosphorylation. However, a minor degree of phosphorylation of these residues cannot be excluded. Then, by means of an *in silico* approach (NetPhos 3.1, http://www.cbs.dtu.dk/services/NetPhos/), we searched for p27 putative phosphorylations induced by G9R substitution. The analysis points to S7 and S12 as possible phosphorylatable sites. The involvement of both residues was investigated by transfection experiments in PC‐3 cells. No important changes in the phosphorylation pattern were observed by expressing S7A/S10A/G9R‐p27 plasmid (Fig. S5A, right). In the same experiment, we also ruled out the involvement of S83 and S183 as possible residues of G9R‐p27 modification (Fig. S5A, right). Conversely, as showed in Fig. 2D, the single substitution of S12 strongly reduces G9R‐p27 phosphorylation, while in S10A/S12A/G9R‐p27 2D‐analysis the signals (1 and 2) for the protein phosphoforms are almost completely abolished. The plot at the bottom shows the occurrence of each isoform as percentage of total for G9R‐p27 and its derivatives, obtained from three independent experiments. The findings suggest that G9R mutation creates a novel strong *consensus site* for S12 phosphorylation (Fig. 2E). Similar results were obtained in additional cell models (Fig. S5B,C). This PTM is specific of G9R‐p27 variant since S12A substitution has no effects on p27 2D pattern (Fig. S5D).

**Fig. 3:**
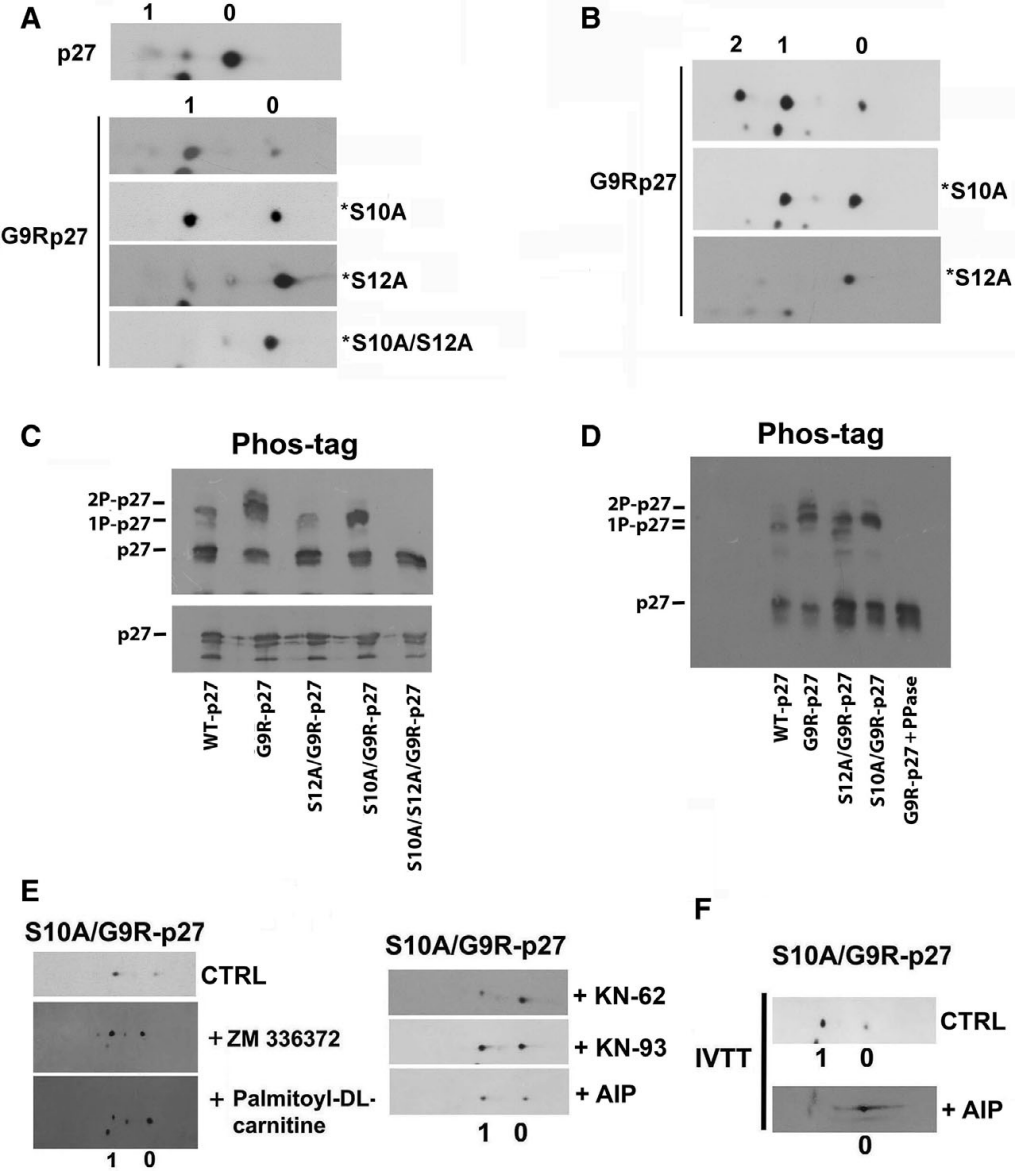
Characterization of G9R‐p27 phosphorylation. (A) pcDNA3.0 plasmids encoding p27, G9R‐p27, S10A/G9R‐p27 (*S10A), S12A/G9R‐p27 (*S12A), and S10A/S12A/G9R‐p27 (*S10A/S12A) were translated and transcribed *in vitro* as reported under Materials and methods. Each IVTT assay mixture was then analyzed by 2D/WB as reported in Figure 2. The filters were analyzed by anti‐p27 mAb. Signals 0 and 1 correspond to unmodified and 1Pi‐proteins, respectively. (B) MEF cells were transfected with three different pcDNA3.0 plasmids encoding G9R‐p27, S10A/G9R‐p27 (*S10A), and S12A/G9R‐p27 (*S12A). After 24‐h transfection, cells were pelleted and cell extracts were prepared as reported under Materials and methods. Equal amounts of protein were analyzed by 2D/WB employing anti‐p27 mAb. Signals 0, 1, and 2 correspond to unmodified, 1Pi‐, and 2Pi‐proteins, respectively. (C) PC‐3 cells were transfected with five different pcDNA3.0 plasmids encoding WT‐p27, G9R‐p27, S12A/G9R‐p27, S10A/G9R‐p27, and S10A/S12A/G9R‐p27. After 24‐h transfection, cells were pelleted and extracts were prepared as reported under Materials and methods. Then, equal amounts of protein were analyzed by 1D/WB (lower image) and SDS/PAGE on polyacrylamide gel strengthened with agarose containing 20 mm Mn^2+^–Phos‐tag (upper image). The filters were analyzed by anti‐p27 mAb. Further details are reported under Materials and methods Section. (D) An experiment similar to that reported in Panel C is showed, except that an aliquot of extract from PC‐3 cells expressing G9R‐p27 (prepared without phosphatase inhibitors) was treated with protein phosphatase lambda (PPase). The samples were then analyzed by Phos‐tag SDS/PAGE, as reported in panel C and in Materials and methods Section. (E) PC‐3 cells were transfected with pcDNA3.0 encoding S10A/G9R‐p27 in order to study only the phosphorylation of G9R‐p27 on S12. Then, cRaf inhibitor (ZN336372), PKC inhibitor (palmitoyl‐DL‐carnitine), and three CAMK II inhibitors (KN‐62, KN‐93, and AIP) were added 2 h before the transfections. After 24 h, cell extracts were prepared and analyzed by 2D/WB employing anti‐p27 mAb compared with cells treated with vehicle as CTRL. The employed concentrations of the compounds are reported in Table 1. (F) pcDNA3.0 encoding S10A/G9R‐p27 plasmid was transcribed and translated *in vitro* in the absence (CTRL) and presence of AIP (CAMKII inhibitor). The mixtures were analyzed by 2D/WB employing anti‐p27 mAb.

To confirm the identification of the phosphorylated residue, a series of *in vitro* transcription and translation (*IVTT*) reactions, employing plasmids encoding wt‐p27, G9R‐p27, S10A/G9R‐p27, S12A/G9R‐p27, and S10A/S12A/G9R‐p27, were performed. Each *IVTT* mixture was analyzed by 2D/WB (Fig. 3A). While wt‐p27 was very scarcely converted in 1P‐form, G9R‐p27 analysis showed two signals corresponding to unmodified protein (signal 0) and to the more abundant 1P derivative (signal 1). Intriguingly, signal 1 was evidenced in S10A/G9R‐p27, but only scarcely detectable or absent in S12A/G9R‐p27 and S10A/S12A/G9R‐p27, respectively. These findings suggest that rabbit reticulocyte extract contains putative G9R‐p27 S12 kinase activity(ies).

We also expressed plasmids encoding G9R‐p27, S10A/G9R‐p27, and S12A/G9R‐p27 in MEFs. Since mouse p27 has a different molecular weight and migrates faster than human p27, MEFs allowed us to identify definitely the 2D/WB signal patterns of the transfected human protein. As shown in Fig. 3B, the obtained data confirm the results reached in human cell lines.

Finally, we employed a phosphate‐affinity gel electrophoresis to further confirm our findings. In this approach, a functional reagent (Phos‐tag) selectively binds phosphorylated proteins and retards their electrophoretic mobility. Figure 3C shows that Phos‐tag/SDS/PAGE analysis of PC‐3 cells expressing wt‐p27, G9R‐p27, S12A/G9R‐p27, S10A/G9R‐p27, and S10A/S12A/G9R‐p27 clearly confirms the data previously obtained. In an additional experiment (Fig. 3D), a sample of protein phosphatase‐treated extract from G9R‐p27‐expressing cells was also analyzed.

The abundance of (pS12)G9R‐p27 in all the cellular models tested suggests that different kinases might recognize the novel generated consensus. To putatively identify the kinase(s) responsible for S12 modification, G9R‐p27‐ or S10A/G9R‐p27‐transfected cells were treated with an array of approximately 50 different specific kinase inhibitors (Table 1). Of the tested compounds, only CaMKII (an arginine‐directed kinase) and, at different extent, cRAF and PKC inhibitors (Fig. 3E) affect the PTM. In addition, a highly specific CaMKII inhibitor (AIP) was directly added to G9R‐p27 *IVTT* assay since, as reported above, rabbit reticulocytes extract contains high G9R‐p27 S12‐kinase activity (Fig. 3F). Although not conclusive, the results allow the exclusion of several kinases as responsible of S12 phosphorylation and suggest that CaMKII might be involved in the investigated modification of G9R‐p27.

In summary, we demonstrated that the cancer‐associated G9R substitution creates a novel consensus sequence for arginine‐directed kinases that allows the quantitative phosphorylation of S12, a residue that is unmodified in wt‐p27. Preliminary data suggest that different protein kinases might be responsible for the identified modifications. At the best of our knowledge, glycine 9‐>arginine change represents the first example of a gene mutation associated with cancer that drives a dramatic hyperphosphorylation on a usually unaffected residue.

### 3.3 Serine 12 phosphorylation causes the loss of p27 tumor suppressor activities

The occurrence of an unexpected and quantitative phosphorylation on S12 residue of G9R‐p27 suggested a possible connection between the novel modification and the loss of p27 anticancer functions.

**Fig. 4:**
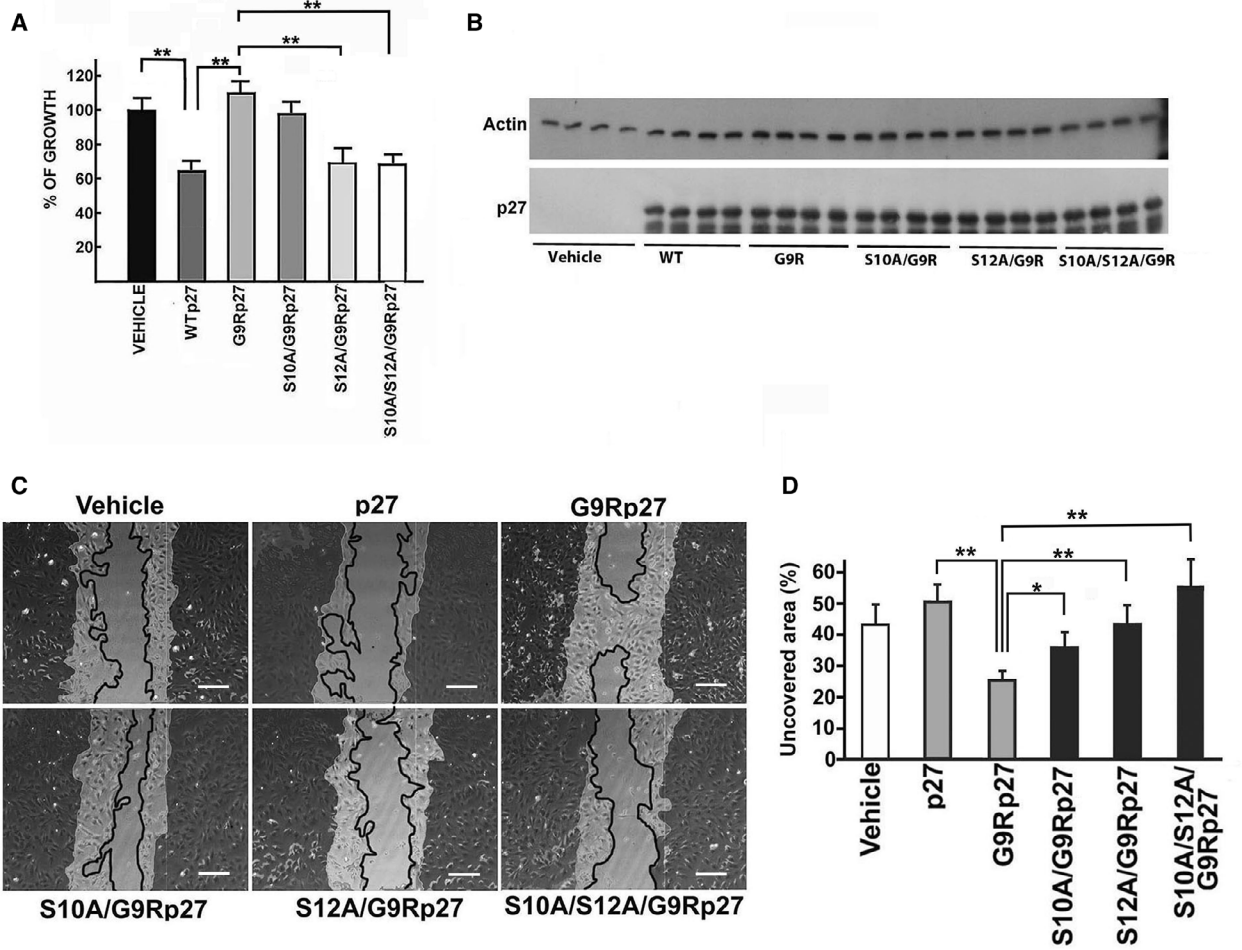
Effect of S12 phosphorylation on the phenotypic activities of G9R‐p27. (A) MEF cells were transfected with different pcDNA3.0 plasmids, namely empty plasmid (indicated as VEHICLE) and plasmids encoding p27 (WTp27), G9R‐p27, S10A/G9R‐p27, S12A/G9R‐p27, and S10A/S12A/G9R‐p27. After 72‐h transfection, the cell number in each well was determined by direct cell counting. Each value is the mean of four independent transfections; bars represent standard deviation. Data were analyzed by Student's *t*‐test. **P < 0.01. (B) MEFs from experiments described in Panel A were used to prepare cell extracts. These extracts were analyzed by WB using anti‐p27 mAb for evaluating p27, G9R‐p27, and its derivatives’ content in the transfected cells. Actin was determined as loading control. (C) The panel shows representative images of a wound‐healing experiment. LN‐229 cells were transfected with the empty pcDNA3.0 vector (Vehicle), and different plasmids as reported in the figure. The darker areas outline the initial wound border, while the continuous line the migration front. Scale bar corresponds to 50 µm. (D) The plot reports the quantitation of the wound‐healing experiment showed in C. Columns represent the percentage of uncovered area by each cell population after 24 h of wound healing (with respect of the uncovered area at time 0), calculated by imagej analysis of the images. Error bars represent the standard deviation of the mean of three independent experiments. Data were analyzed by Student's *t*‐test. *P < 0.05; **P < 0.01.

Thus, the growth rate of MEFs expressing wt‐27, G9R‐p27, S10A/G9R‐p27, S12A/G9R‐p27, and S10A/S12A/G9R‐p27 was investigated (Fig. 4A). The results suggested that the abrogation of S12 phosphorylatable site (i.e., the expression of S12A/G9R‐p27 and S10A/S12A/G9R‐p27) resulted in an almost complete rescue of p27 growth inhibitory activity. Conversely, the transfection of S10A/G9R‐p27, similarly to G9R‐p27, did not affect the proliferation rate compared with the control transfected with empty vector (Vehicle). Equal expression levels of the proteins were also demonstrated (Fig. 4B).

Although controversies exist in the literature, it is undoubted that p27 plays crucial functions in cytoskeleton rearrangement and cell migration [11, 12, 13, 14, 15, 16, 17, 18].

To investigate the effects of p27 mutation on cell movement, we compared the migration of LN‐229 cells transfected with wt‐p27 and G9R‐p27 by means of a wound‐healing assay. Identically sized wounds were generated in confluent monolayers of control (i.e., transfected with the empty vector), wt‐p27‐, and G9R‐p27‐expressing cells, and the wound‐healing process was periodically monitored. The cells were cultured in serum‐deprived medium for minimizing the effects of cell proliferation on the motility evaluation. As shown in Fig. 4C,D, the cell‐free gap area in wt‐p27‐expressing cells was higher compared with control cells; by contrast, G9R‐p27 expression resulted in an increased healing, thus indicating that the residue substitution causes an effect on cell motility opposite to that of wt‐p27. Importantly, the analysis of S10A/G9R‐p27, S12A/G9R‐p27, and S10/S12A/G9R‐p27 mutants confirmed the role of S12 phosphorylation in G9R‐p27‐dependent increase in cell motility (Fig. 4C,D).

**Fig. 5:**
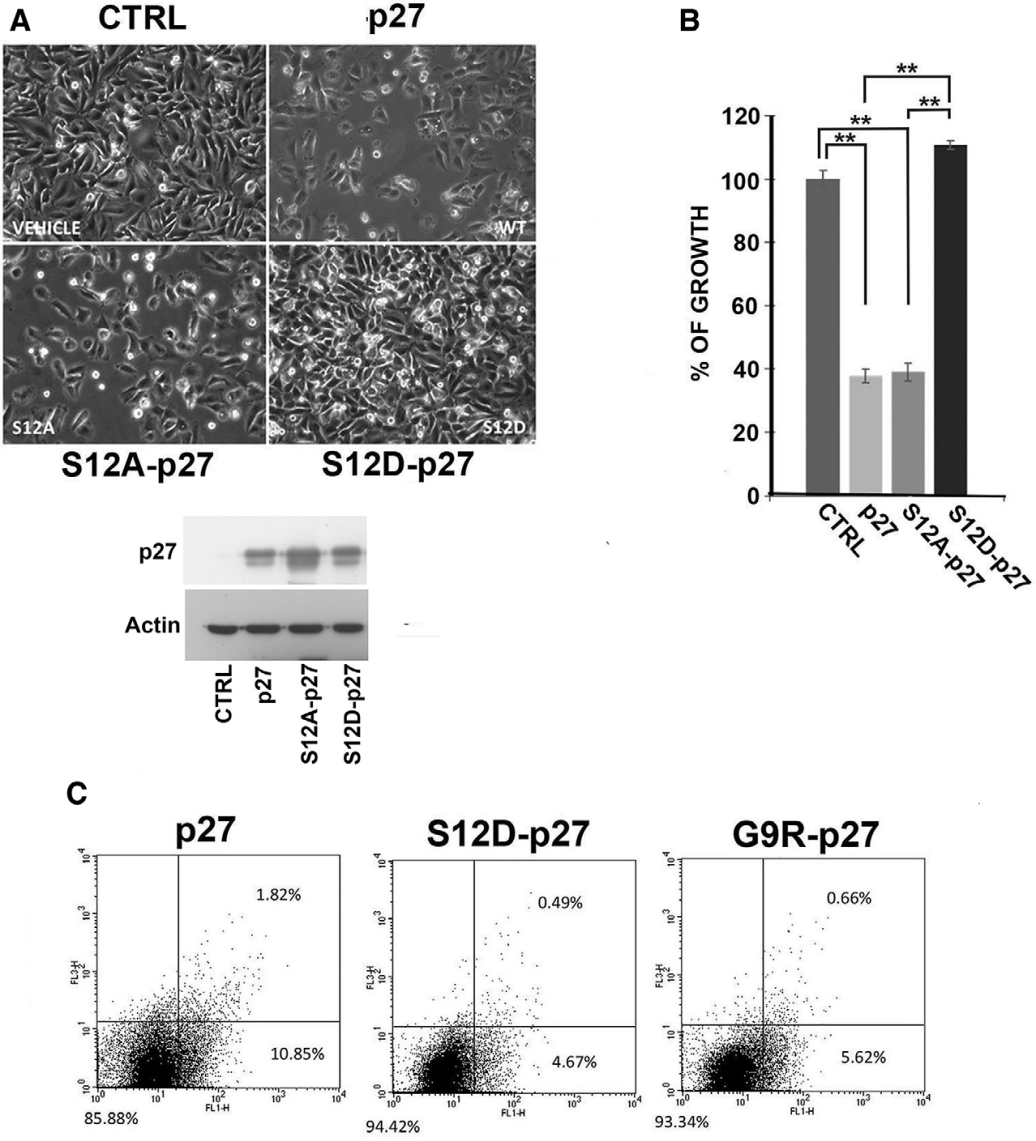
Role of serine 12 phosphorylation in the loss of p27 tumor suppressor activities. (A) Effects of mutations on p27 protein affecting S12, specifically S12A and the phosphomimetic S12D, on PC3 proliferation were evaluated. Empty vector (CTRL), and plasmids encoding p27, S12A‐p27, and S12D‐p27 were transfected into HEK293 cells. After 72‐h incubation, microscopy images were taken (10×). At the bottom, transfection efficiency was confirmed by WB using anti‐p27mAb. Actin was analyzed as loading control. (B) The graph reports the results of direct cell counting for the experiments shown in A. Columns represent the mean of three replicates as in Panel A and bars the standard deviation. Data were analyzed by Student's *t*‐test. **P < 0.01. (C) PC3 cells were transfected for 48 h with pcDNA3.0 plasmid encoding p27, S12D‐p27, and G9R‐p27, followed by staining with Alexa Fluor 488 Annexin V FITC. Apoptosis was evaluated by flow cytometry and calculated from 20 000 events. Right lower quadrant comprises cells that are stained only with Annexin V FITC, therefore cells in early stage of apoptosis; and in right upper quadrant cells stained with both Annexin V FITC and propidium iodide are comprised, representing cells in late apoptosis. Left lower quadrant comprises nonapoptotic cells. Each cell population is expressed as percentage of total analyzed events.

To corroborate the relevance of S12 phosphorylation, we prepared a p27 phosphomimetic in which serine 12 was modified into aspartate, namely S12D‐p27. In addition, S12Ap27‐encoding vector was also used to evaluate whether the removal of serine 12 could specifically affect the activity of p27. Thus, empty vector and plasmids encoding wt‐p27, S12A‐p27, and S12D‐p27 were transfected into PC‐3 cells. As shown in Fig. 5A, while wt‐p27 and S12A‐p27 strongly inhibit cell growth, S12D‐p27 was unable to affect the proliferation. Figure 5B reports the results of three independent experiments of transfection as in Fig. 5A. The transfection efficiency and comparable levels of the expressed proteins were confirmed by immunoblotting (Fig. 5B).

Subsequently, the capability of inducing apoptosis of S12D‐p27 was compared to that of p27 and of G9R‐p27. The experiment was performed similarly to that reported in Fig. 1E except that transfection was for 48 h. As shown in Fig 5C, cells expressing the phosphomimetic S12D‐p27 protein had a reduced rate of apoptosis compared with those expressing wt‐p27, at an extent comparable to that of G9R, thus confirming the role of this specific phosphorylation in modulating the activity of the p27 variant.

In summary, the phosphorylation of S12 appears to be responsible for the loss of anticancer activity of GR9‐p27. Conversely, the change of glycine 9 into arginine does not appear able to affect by itself the activity of the protein, in that G9R‐p27 mutants lacking S12 phosphorylation (i.e., S12A mutants) show normal antiproliferative function. The relevance of S12 phosphorylation is strongly confirmed by S12D‐p27 behavior.

### 3.4 Serine 12 phosphorylation alters G9R‐p27 localization and CDK interaction

**Fig. 6:**
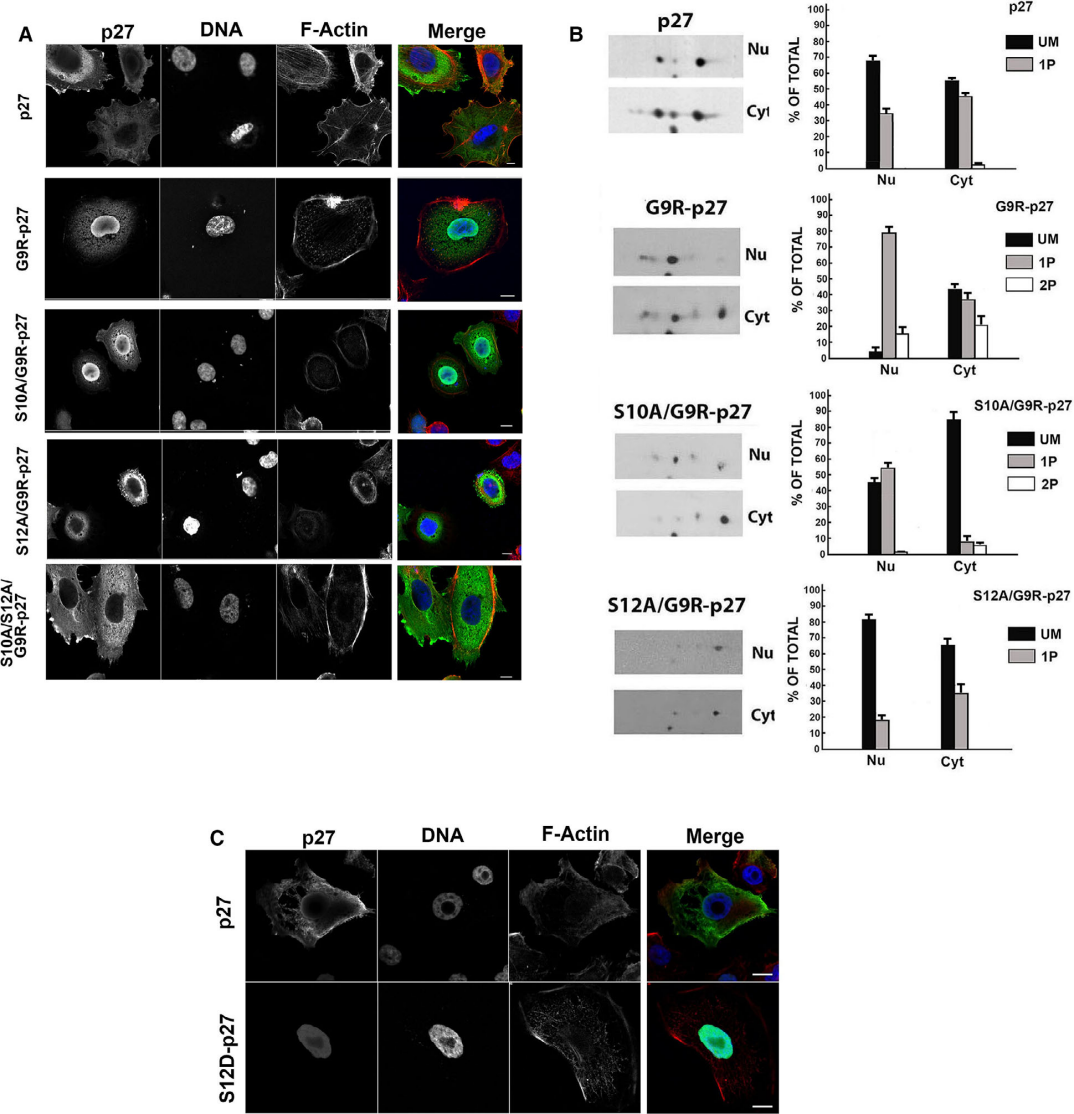
Cellular localization of G9R‐p27 phosphoisoforms. (A) Cellular localization of p27 and G9R‐p27 mutants was investigated by immunofluorescence. Representative images of PC‐3 cells transfected with pcDNA3.0 plasmids encoding p27, G9R‐p27, S10A/G9R‐p27, S12A/G9R‐p27, and S10A/S12A/G9R‐p27. Cells were stained with Hoechst 33342 (DNA, blue), phalloidin, (F‐actin, red), and anti‐p27 Ab (p27, green). Images were visualized using a Zeiss LSM510 confocal laser scanning microscope (*n* = 3). Typical images acquired by confocal microscopy are shown. A 63× objective was used. Further details are under ‘Materials and methods’ Section. Scale bar corresponds to 10 µm. (B) PC‐3 cells were transfected with pcDNA3.0 encoding p27, G9R‐p27, S10A/G9R‐p27, and S12/G9R‐p27. After 24 h of incubation, nuclear and cytosol compartments were prepared and analyzed by 2D/WB. On the left, results of 2D/WB using anti‐p27 mAb are shown. Further details are reported under Materials and methods. On the right, the plot reports the percentage of the relative signal intensity associated with a single spot in respect of the total for each 2D analysis shown in panel A. Nu, nuclear fraction; Cyt, cytoplasmic extract. (C) PC‐3 cells were transfected with pcDNA3.0 plasmids encoding p27 and S12D‐p27. Then, the cells were stained with Hoechst 33342 (nuclear staining, blue), phalloidin, (actin, red), and Alexa Fluor 488 (p27, green). Images were visualized at a confocal laser scanning microscope as in panel A [*n* = 3]. A 63× objective was used. Scale bar corresponds to 10 µm.

In the primary parathyroid adenoma specimen of the patient with the *CDKN1B* heterozygous mutation (c. 25C>A, G9R‐p27), immunohistochemistry showed that p27 signal accumulated in the nuclear compartment [51]. Thus, to determine whether S12 phosphorylation might impact on G9R‐p27 cell localization, the subcellular distribution of the mutated protein was investigated by immunofluorescence and confocal microscopy. As showed in Fig. 6A, wt‐p27 transfected in PC‐3 cells appears prevalently cytosolic. G9R‐p27, on the other hand, mostly localizes in the nuclei, confirming the data observed in cancer specimens [51]. Intriguingly, the substitution of serine 12 with alanine results in a relocalization of the transfected S12A/G9R‐p27 in the cytoplasm. Conversely, S10A/G9R‐p27 presented a nuclear localization and S10A/S12A/G9R‐p27 was equally distributed between the nuclear and cytosolic compartments. In brief, S12 phosphorylation favors G9R‐p27 nuclear accumulation, while S10 phosphorylation is required for its cytosolic localization, as also reported for wt‐p27 [37].

The confocal microscopy data were then corroborated by biochemical studies aimed at evaluating the nuclear and cytosol distribution of the different isoforms of G9R‐p27 variant and of its derivatives (S10A/G9R‐p27, S12A/G9R‐p27) compared with wt‐p27 employing 2D/WB. A shown in Fig. 6B, nuclear extracts of G9R‐p27‐transfected PC3 cells contained mainly 1P and 2P isoforms, but scarce levels of the unmodified form. Conversely, the cytosol includes mostly the unmodified and the 1P‐isoforms of G9R‐p27 (Fig. 6B, 2D/WBs on the left and histograms obtained by the densitometric analysis of 2D spots on the right). The analysis of S10A/G9R‐p27 clearly shows that the mono‐pS12‐derivative is mostly confined at nuclear level, while S12A/G9R‐p27, that presents a phosphorylation only on S10, has the 1P‐isoform mainly in the cytosolic compartment (Fig. 6B, 2D/WBs and histograms). Thus, 2D/WB analyses sustain the importance of S12 phosphorylation for the localization of G9R‐p27 mutant at nuclear level. The relevance of the modification in the nuclear compartmentalization of the protein is strongly confirmed by the immunofluorescence analysis of S12D‐p27 cellular distribution (Fig. 6C).

Nuclear localization of G9R‐p27 might be due to different mechanisms. It has been reported that p27 S10 phosphorylation is required for nuclear shuttling since it allows the binding of the CKI with CRM1, a cargo protein [37, 38]. Thus, S12 phosphorylation could potentially affect G9R‐p27 CRM1 interaction, thus causing G9R‐p27 nuclear accumulation. However, the cytosolic relocalization of S10A/S12A/G9R‐p27 does not argue in favor of this hypothesis.

Alternatively, it is conceivable that S12 modification influences the binding of G9R‐p27 with the cyclin‐CDK complexes, and this might be responsible for, at least in part, the mutant subcellular localization. To evaluate this hypothesis, we transfected G9R‐p27 into MEFs lacking either CDK2 or CDK4 or both CDK2/CDK4. The absence of the kinases was confirmed by immunoblotting (Fig. S6A). As reported in Fig. S6B, G9R‐p27 shows a reduced nuclear/cytosolic level ratio in CDK2^−/−^ and CDK4^−/−^ MEFs compared with the parental cells. A further nuclear reduction in G9R‐p27 was observed in CDK2^−/−^/CDK4^−/−^ MEFs, suggesting that these kinases play a role in the nuclear localization of the mutant CDK inhibitor. Nuclear extracts of wt‐p27‐ and G9R‐p27‐expressing WT‐MEFs and CDK2^−/−^/CDK4^−/−^ MEFs were then analyzed by 2D‐WB. The results reported in Fig. S6C demonstrated that CDK4/CDK2 absence reverts the preferential nuclear localization of the 1P‐form of G9R‐p27 while all the isoforms (0, 1, and 2) are observable at comparable levels of expression. Scarce effects were, on the other hand, observed on the cytosolic fractions.

In summary, various approaches suggested that S12 phosphorylation plays an important role in G9R‐p27 nuclear localization. In addition, particularly CDK2 but also CDK4 proteins appear involved in this p27 variant compartmentalization.

### 3.5 Interaction of G9R‐p27 with CDKs

To reconcile the nuclear accumulation of G9R‐p27 with the absence of antiproliferative effects, we focused our interest on the interaction of the mutant protein with nuclear CDKs.

**Fig. 7:**
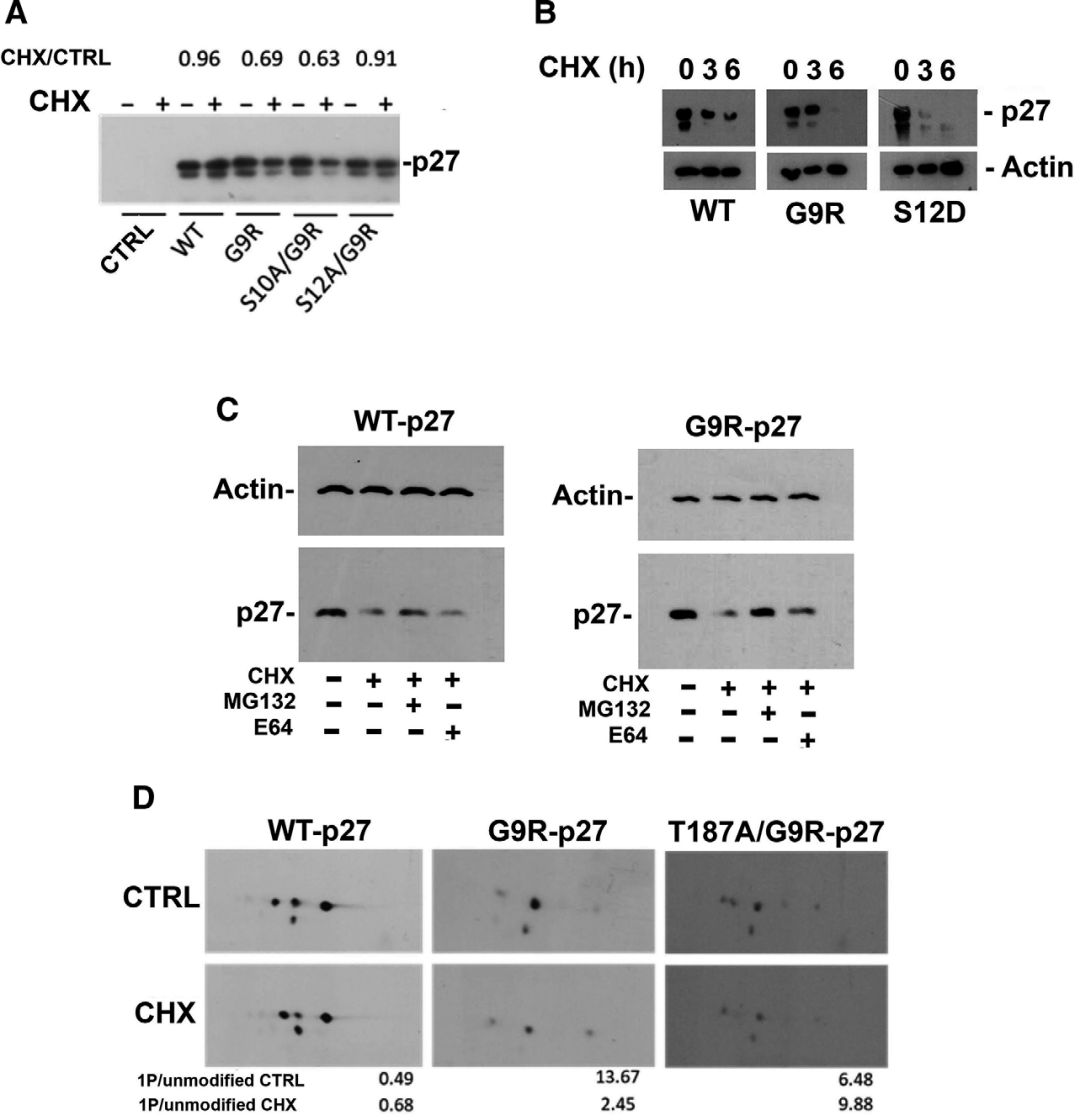
Effect of G9R mutation on p27 removal. (A) PC‐3 cells were transfected with pcDNA3.0 empty vector (CTRL), and pcDNA3.0 encoding p27 [WT], or G9R‐p27 [G9R], S10A/G9R‐p27 [S10A/G9R], and S12A/G9R‐p27 [S12A/G9R]. After 18‐h transfection, medium was changed and cells were treated with or without CHX (36 µm) for 6 h. After harvesting, cells were lysed and analyzed by western blotting using anti‐p27 mAb. The intensity of the specific signals was evaluated using imagej software, and value of the relative intensity over the CTRL (+CHX/−CHX) +CHX/CTRL is reported for each protein under analysis. (B) K562 cells were transfected with pcDNA3.0 encoding p27 (WT), G9R‐p27 (G9R), or S12D‐p27 (S12D). After 18‐h transfection, medium was changed and cells were treated with CHX (36 µm) for 0, 3, or 6 h. After harvesting, cells were lysed and analyzed by western blotting using anti‐p27 mAb. Actin was used as loading control. (C) K562 cells were transfected with pcDNA3.0 encoding p27 (WT) and G9R‐p27 (G9R). After 18‐h transfection, medium was changed and cells were treated as reported in the panel. After harvesting, cells were lysed and analyzed by western blotting using anti‐p27 mAb. Actin was used as loading control. (D) PC‐3 cells were transfected with pcDNA3.0 encoding p27 [WT], or G9R‐p27 [G9R], T187A/G9R‐p27 [T187A/G9R]. After 18‐h transfection, medium was changed and cells were treated with or without CHX (36 µm) for 6 h. After harvesting, cells were lysed and analyzed by 2D‐WB using anti‐p27 mAb. The intensity of the specific signals was evaluated using TotalLab CLIQS gel image analysis Software and the relative intensities of the monophosphorylated isoform [1P] over the unmodified isoform for each analysis are reported in the histograms at the bottom of the panel.

Initially, we evaluated in PC‐3 cells the effect of CHX (an inhibitor of protein synthesis), at 36 µm concentration, on the removal of p27, G9R‐p27, and its S10A and S12A derivatives. The putative decrease in the protein was evaluated after 6‐h incubation to evidentiate the first phase of removal. As shown in Fig. 7A, G9R‐p27 is more rapidly degraded compared with wt‐p27. The minor stability appeared related to S12 phosphorylation, since the abrogation of S12 phosphorylatable site (S12A/G9R‐p27) reverts the effect. To corroborate the finding, a time course of CHX treatment is reported in Fig. 7B. The experiment was performed in K562 cells, to facilitate an equal withdrawal of cells. Also in this case, G9R‐p27 exhibits a shorter half‐life compared to wt‐27. Most interestingly, the analysis of S12D‐p27 shows that the protein is almost completely degraded at 3‐h incubation, pointing to S12 phosphorylation as a key event in the control of the cellular levels of G9R‐p27. Furthermore, we evaluated the activity of MG132, a specific inhibitor of the proteasome‐dependent degradation, compared to the effect of E64, that, differently, interferes with the lysosomal proteases. As shown in Fig. 7C, the inhibition of the proteasome activity stabilizes wt‐p27, as expected, and even more G9R‐p27, indicating that the accelerated removal of G9R‐p27 does involve the proteasome‐dependent degradation. Differently from the wt‐counterpart, the mutant levels are slightly increased under E64 treatment. This finding is suggestive of a partial lysosomal protease involvement in G9R‐p27 degradation.

Finally, to recapitulate the mechanism of G9R‐p27 degradation, we investigated the 2D‐WB pattern of WT‐p27, G9R‐p27, and T187A/G9R‐p27 before and after 6 h of 36 µm CHX treatment (Fig. 7D). T187A/G9R‐p27 was employed to understand the role of T187 phosphorylation in the degradation process. The obtained results suggested that the reduction in G9R‐p27 is related to an increase in instability of the monophosphorylated G9R‐p27 form, mainly dependent on T187 role.

Considering the importance of phosphorylation of T187 in p27 and in G9R‐27 removal, we concentrated on CDK2 and CDK1, which are the kinases responsible for this modification. Indeed, the inhibition of these two kinases represents a vital mechanism of p27‐dependent growth arrest.

**Fig. 8:**
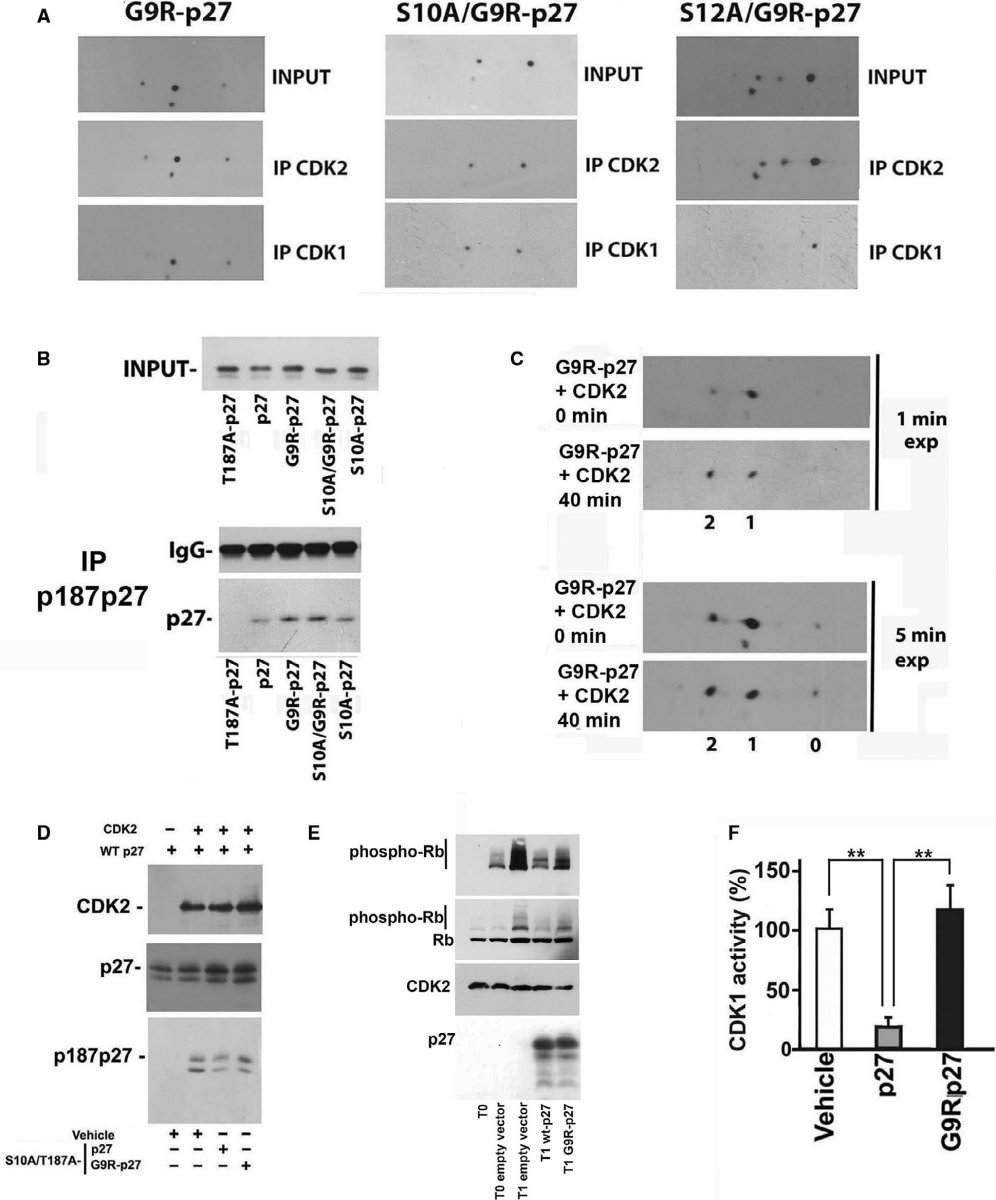
Effect of G9R‐p27 on CDK activities and putative identification of G9R‐p27 S12 kinases. (A) PC‐3 cells were transfected with pcDNA3.0 plasmids encoding G9R‐p27, S10A/G9R‐p27, and S12A/G9R‐p27. The nuclear compartments were purified and immunoprecipitated with rabbit pAb against CDK1 and CDK2. Each set of IPs was analyzed by 2D/WB, together with input extract. From left to right, 2D/WB analyses for G9R‐p27, S10A/G9R‐p27, and S12A/G9R‐p27 transfected cells are shown. Further details are reported under ‘Materials and methods’. (B) PC‐3 cells were transfected with pcDNA3.0 plasmids encoding T187A/p27, p27, G9R‐p27, S10A/G9R‐p27, and S10A‐p27. After 24‐h transfection, cells were treated with Mg132 (a proteasome inhibitor) for additional 8 h. Subsequently, nuclear extracts were prepared and immunoprecipitated with anti‐p187p27 rabbit pAb. The IP was then analyzed by western blotting employing anti‐p27 mAb. Extract from T187A/p27‐expressing cells was used to confirm the specificity of anti‐p187p27 pAb. On the top, input extracts were evaluated. On the bottom, the IP materials were tested for IgG and p27 content. Further details are reported under Materials and methods. (C) G9R‐p27 protein was prepared from PC‐3‐transfected cells. The partially purified protein was incubated with active human recombinant CDK2/CycE‐GST enzyme. Then, the assay mixtures at time zero and after 40 min of incubation were analyzed by 2D/WB. The films were exposed for different time periods to support the conclusions reached in the text. Further details are reported under Materials and methods. (D) Partially purified dephosphorylated human WT‐p27 was incubated with active human recombinant CDK2/CycE‐GST enzyme in the presence of IVTT S10A/T187A‐p27 or S10A/T187A‐G9R‐p27, to compare the inhibition activity of these latter proteins. The abrogation of the phosphorylatable site in T187 and in S10 (S10A/T187A mutants) was introduced to avoid the recognition of these sites as substrate in the *in vitro* assay of CDK2. Vehicle represents product of IVTT reaction carried in the presence of empty vector. Anti‐p187p27 rabbit pAb was used to evaluate CDK2 activity on human recombinant WT‐p27 protein. Anti‐p27 and anti‐CDK2 antibodies were used as controls of the experiment. (E) CDK2 assay determination was performed employing human recombinant active CyclinE/CDK2 and Rb as substrate. The first lane from left (T0, zero time) corresponds to a control assay, that is, a mixture including CyclinE/CDK2 and Rb but not incubated at 30 °C. T0 empty vector (second lane) is a control assay (i.e., a not incubated assay) that includes CyclinE/CDK2, Rb and a PC‐3 cell extract transfected with a pcDNA3.0 empty vector. All T1 lanes correspond to assay reactions incubated for 1 h at 30 °C. In particular: T1 empty vector mixture includes Rb, CyclinE/CDK2, and PC‐3 cell extract transfected with a pcDNA3.0 empty vector; T1 wt‐p27 includes Rb, CyclinE/CDK2, and PC‐3 cell extract transfected with a vector encoding wt‐p27, and T1 G9R‐p27 includes Rb, CyclinE/CDK2, and PC‐3 cell extract transfected with a vector encoding G9R‐p27. Additional details are reported under Materials and methods. The assay reactions were analyzed by 1D/WB employing antibodies against phospho‐Rb, Rb, CDK2, and p27. (F) CDK1 assay determination was performed employing ADP‐Glo Kinase Assay Kit and human recombinant active CDK1/Cyclin A2. The reaction mixtures included (or did not include, Vehicle) partially purified wt‐p27 or G9R‐p27. Error bars represent the standard error of the mean of three independent experiments. Data were analyzed by Student's *t*‐test. **P < 0.01.

Then, we investigated the association between G9R‐p27 (and its S10A and S12A derivatives) and CDKs by IP‐2D/WB. As shown in Fig. 8A, CDK2 associates with all G9R‐p27 isoforms and the bidimensional pattern of anti‐CDK2 immunoprecipitated G9R‐p27 roughly reproduces that of the input extract. Conversely, CDK1 immunoprecipitations show that the unmodified and pS12G9R‐p27 (1P‐isoform) interact with the kinase, while pS10G9R‐p27 derivatives are unable to do it (1P‐isoform in S12A/G9R‐p27 pattern). This finding is in accord with our previous data showing that pS10p27 does not bind the CDK1 containing complexes [40].

Although all the isoforms of G9R‐p27 appear to interact equally with CDK2, this does not necessarily correspond to effective CDK2 inhibition. Thus, we first evaluated the ability of G9R‐p27 to act as CDK2 substrate, by immunoprecipitation experiments using anti‐pT187p27 antibodies in PC‐3 cells expressing different mutants. The cells were treated with MG132 to avoid phosphoT187‐dependent degradation and to estimate only the ability of being modified.

From this experiment, we concluded that G9R‐p27 is phosphorylated on T187 by CDK2 *in vivo* at an extent higher than that of wt‐p27 (Fig. 8B). In addition, the substitution of S10 with alanine did not alter T187 phosphorylation, suggesting that S10 phosphorylation is not required for T187 modification (Fig. 8B). The results also suggested that G9R‐p27 (or its derivatives) inhibits CDK2 at a lower extent.

A further detailed characterization of G9R‐p27 phosphorylation by CDK2 is a difficult matter to investigate since the phosphorylation of each phosphoisoforms should be evaluated. Therefore, a G9R‐p27 preparation (comprehensive of all the phosphoisoforms) was partially purified from transfected cells and employed as substrate in an *in vitro* kinase assay using recombinant CDK2. The assay mixture was then analyzed by 2D/WB. Preliminary, we employed S10A/S12A/T187A/G9R‐p27 as CDK2 substrate excluding the existence of CDK2‐phosphorylatable G9R‐p27 residues in addition to S10, S12, and T187 (Fig. S6D). Figure 8C shows the results of assaying partially purified G9R‐p27 as CDK2 substrate. First, it is clear that a large amount of 1P‐isoform was converted in 2P‐isoform. Conversely, the 2P isoform was not converted in a 3P derivative (i.e., pS10/pS12/G9R‐p27 is not phosphorylated in T187). Intriguingly, also the unmodified G9R‐p27 signal did not change its levels. The finding was repeatedly obtained in several independent experiments. We concluded that probably G9R‐p27 monophosphorylation favors the mutant ability to act as CDK2 substrate. The results confirmed the finding reported in Fig. 6B.

To further demonstrate that G9R‐p27 mutant does not efficiently inhibit the CDK2 kinase activity, we performed an *in vitro* assay for CDK2 activity using partially purified human wt‐p27 itself as substrate and immunoblotting for pT187p27 as detection method. Equal amount of IVTT‐obtained wt27 and G9R‐p27, both bearing S10A and T187A mutations (to avoid the capability of being phosphorylated on these two residues), were preincubated with recombinant active CDK2 complex. Then, CDK2 complex was used to phosphorylate hr‐p27. The activity was evaluated by detecting T187 phosphorylation. As shown in Fig. 8D, while S10A/T187A‐p27 addition resulted in a decrease in the specific pT187‐p27 signal, S10A/T187A/G9R‐p27 had no inhibitory effect on T187 phosphorylation of wt‐p27 substrate. The decreased G9R‐p27 inhibitory activity on CDK2 was also confirmed by employing Rb protein as substrate. As shown in Fig. 8E, while wt‐p27 almost completely inhibits the enzymatic activity, G9R‐p27 exerts about 50% inhibition.

Finally, we also tested the inhibitory effect of wt‐p27 and G9R‐p27 on CDK1 activity, employing histone H1 as substrate. While wt‐p27 exerted a remarkable inhibition, G9R‐p27 did not affect the kinase activity (Fig. 8F).

Conclusively, the results reported suggest that G9R‐p27 interacts with CDK2 but scarcely reduces its enzymatic activity as well as the capability of the kinase to modify wt‐p27 or G9R‐p27 itself. Moreover, unmodified G9R‐p27 and pS12G9R‐p27 (differently from pS10G9R‐p27) bind but do not inhibit CDK1.

## 4 Discussion

The awareness that *CDKN1B* haploinsufficiency is involved in cancer development and evolution has become progressively more stringent, thus making *CDKN1B* a major example of haploinsufficient TSG [64].

In the past, a decrease in p27 levels or its cytosolic mislocalization has been demonstrated in a large percentage of different human cancers, frequently associated with poor prognosis and survival [29, 44]. These events have been generally explained by an accelerated p27 removal or an altered phosphorylation on known residue. In some cases, germinal or acquired abnormalities of 12p13 chromosome, where *CDKN1B* maps, have been described ([29] and reference therein). Frameshift and nonsense mutations occur and result in p27 protein truncation. This impacts on the stability of the protein, on its localization, or may result in the defeat of the C‐terminal‐associated functions. *CDKN1B* missense mutations have been only recently reported and suggested as cause of p27 haploinsufficiency. The genetic alterations have been identified in various human cancers including the following: neuroendocrine tumors, breast, prostate, thyroid, and parathyroid cancers [29, 53]. However, the mechanisms by which *CDKN1B* missense mutations might induce cancer development or haploinsufficiency remain largely obscure. Accordingly, we focused our attention on these genetic changes, since they might also furnish additional and unprecedented information on the structural requirements for p27 tumor suppressor functions.

Here, we report a study on the effect of c.25A>C (pG9R) *CDKN1B* substitution, a germinal mutation identified in heterozygosity in a parathyroid adenoma seen as MEN‐related syndrome [51]. The results obtained disclose a novel mechanism for inactivating TSG.

We evaluated the phenotypical effect of the mutation by transfecting either wt‐p27 or G9R‐p27 in human cell lines. While p27 overexpression led to an expected inhibition of proliferation and to apoptosis activation, G9R‐p27 did not show these effects. The findings were obtained in different cell models and with similar levels of transfected protein. Analogously, G9R‐p27‐expressing cells loss the capability of reducing transwell migration that was, conversely, observed in p27‐transfected cells. Repeated experiments demonstrated that G9R‐p27 enhances (with respect of control and p27‐overexpressing cells) the ability of glioma cells to form spheres in soft medium (see images in Figs 1D and S2). This unanticipated result might be due either to the loss of endogenous p27 functions or to the acquirement of G9R‐p27 novel activities/interactions or both the effects. All these data clearly indicate that the missense mutation affects important functions of p27 in determining cell phenotype and behavior.

Being p27 an IUP, we focused our attention on G9R‐p27 PTMs. We observed, in all cell models tested, that the mutation strongly increased protein phosphorylation, with the majority of the protein occurring as monophorylated isoform(s) and a still significantly relevant quantity being a 2P mutant. This pattern is remarkably different from that observed in the wild‐type protein that generally shows a large percentage of protein in a nonmodified status, a minor amount of monophosphorylated isoform and a scarcely detectable (or absent) biphosphoform. Subsequent investigations demonstrated that G9R‐p27 is mostly phosphorylated on S12, a residue not modified in wt‐p27. Mechanistically, the phosphorylation can be explained with the position of S12 that occurs at +3 from the introduced arginine. In other words, the missense G9R mutation creates a powerful novel consensus motif (mainly for basophilic kinases) that allows quantitative S12 phosphorylation [65]. To the best of our knowledge, this is one of the first examples in which a missense mutation determines a phosphorylation on a residue not only distinct from the mutated amino acid but, more intriguingly, also not physiologically modified. To note, the novel PTM is extremely abundant both in transfected cells and in *IVTT* reaction, suggesting, indirectly, that several kinases might phosphorylate G9R‐p27 on S12. By a series of different approaches, we also demonstrated that S12 phosphorylation, and not G9R change, is responsible for the properties of the p27 variant. First of all, S12A/G9R‐p27 essentially behaves as wt‐p27. Second, the relevance of S12 phosphorylation was convincingly confirmed by the effect of the phosphomimetic S12D‐p27 that resembles G9R‐p27 although lacking G9R mutation. This represents a critical aspect of the study which rules out the possibility that the introduction of a bulky and positively charged amino acid (i.e., arginine) in place of a small neutral one might affects the acquired conformation of p27 in different conditions and cellular environment/interactions, thereby resulting in different protein properties and activities. Interestingly, COSMIC database annotates a different somatic mutation affecting glycine 9, specifically G9W‐p27 (c.25G>T) substitution identified in a primary specimen of bladder carcinoma (https://cancer.sanger.ac.uk/cosmic/mutation/overview?id=169908424). However, *in silico* studies on the possible output of G9W mutation indicate that the substitution of glycine in tryptophan does not increase the score for S12 phosphorylation, while it reduces of about 50% the disorder grade of the region. Differently, G9R mutation has no predicted effect on the level of disordered structure of p27. We might speculate that G9W substitution, acting on the flexibility of the region, might affect the promiscuity of the protein, its interaction with kinases responsible for the adjacent serine 10 residue phosphorylation or KID function and specificity.

Our results on G9R variant, instead, point to the novel phosphorylation on S12, and not to G9R substitution, as the causative mechanism of p27 functional changes. Future studies will be devoted to further evaluate the importance of this residue phosphorylation on the structure/interaction/function of p27.

G9R‐p27 (or most probably, its phosphoS12 derivatives) appears to increase cell motility as suggested by wound‐healing experiments. A mechanistic hypothesis is that this could cause a diminished cell‐to‐cell adhesion. A decreased interaction between the cells could also be the basis of the large spheres formed in LN‐229 G9R‐p27‐transfected cells and of the observed cell detachment from the spheres. The increased motility and decreased cell‐to‐cell attachment, in turn, could be associated with the relocalization of G9R‐p27 (confirmed by S12D‐p27) in the nucleus (see confocal images), heavily affecting the cytoplasmic functions of the protein. As a matter of fact, cytosolic p27 is essential for the control of cytoskeleton dynamics and cell motility, and participates, modulating microtubule stability, to the regulation of the H‐Ras endocytic trafficking and ubiquitination, thus affecting cell division [55]. Finally, the apparent inconsistency between nuclear localization and loss of antiproliferative activity is explained by the occurrence of G9R‐p27 (and its pS12 derivatives) binding to CDKs and the scarcity of inhibitory activity.

A possible caveat of this study is that it is essentially based on the transfection of mutant constructs. However, this strategy allowed us to evaluate precisely the role of several p27 residue modifications. Although this approach might affect a direct translation of the experimental data to the *in vivo* conditions, the conclusions reached open promising and unpredictable perspectives. First, we demonstrate that a missense mutation might generate a novel and strong kinase consensus sequence that, when placed at an optimal distance from a phosphorylatable residue (S, T, or Y), might result into a massive unexpected PTM. The novel PTM might directly abolish the tumor suppressor function of a protein and, in our case, cause functional p27 haploinsufficiency or even a gain of unexpected activities. Second, the loss of G9R‐p27 tumor suppressor function includes the lack of CDK inhibition. This might increase CDK activity resulting, *in vivo*, in p27 decrease. Intriguingly, this view is confirmed by the immunohistochemical data in the original cancer specimen that report a reduction of p27 signal and its nuclear accumulation, a pattern compatible with our G9R‐p27 nuclear localization [51]. Third, the identified mechanism provides a working hypothesis for explaining how a germinal genetic variant of a TSG might result in specific tissue patterns of cancers. It appears indeed conceivable that the tissues with high kinase activity(ies) responsible for the novel residue phosphorylation might represent the preferred sites for both tumor suppressor protein loss of function and cancer development.

## 5 Conclusions

The mechanism reported here is, at the best of our knowledge, unique and not previously described. It is important to emphasize that, from now on, particular consideration should be taken when a missense mutation introduces a basic residue (arginine or lysine) located upstream (from +3 to +5) a serine or a threonine, since the direct outcome of the substitution could be the induction of novel PTMs with unexpected protumorigenic properties.

## Conflict of interest

The authors declare no conflict of interest.

## Author contributions

DB, FDR, and AB designed the experiments of this study. DB, ES, AA, AT, CB, and AN conducted the experiments. DB, FDR, and AB performed data analysis and critical discussion of the results. DB, DR, SP, FDR, and AB contributed to the writing and editing of the manuscript. All authors approved the final draft of the manuscript.

## Supporting information


**Fig. S1.** Transfection efficiency evaluation. HEK‐293 cells transfected for 48 h with empy‐vector (CTRL), wt‐p27 and G9R‐p27 were stained with anti‐p27 mAb and fluorescence‐tagged secondary antibodies and analyzed by flow cytometry using a FACScalibur. Calculations were done over 30 000 events. M2 includes wt‐ and mutated p27 expressing cells, corresponding at least to 50% of the whole cell populations. M1 comprises cells with a very low level of fluorescence corresponding to endogenous p27 staining.

**Fig. S2.** Spheroid formation ability of cells expressing wt‐p27 and G9R‐p27. LN‐229 glioblastoma cells transfected the day before with empty vector (CTRL) or plasmids encoding WT‐, and G9R‐p27 were seeded in matrigel for 3D spheroid‐based tumor invasion assay. Details are reported under ‘Materials and methods’. Cultures were observed under light microscope and images were taken at 4, 5 and 6 days after seeding. The experiment was repeated three times, while the figure reports the results of two replicates for each time point. On the right, images obtained after 6 days inclusion at higher magnification: G9R‐expressing cultures show the presence of cells that appear detaching from spheres (protruding cells).

**Fig. S3.** Spheroid formation ability of PC‐3 cells expressing wt‐p27 and G9R‐p27. PC‐3 cells transfected the day before with empty vector (CTRL) or plasmids encoding WT‐, and G9R‐p27 were seeded in matrigel for 3D spheroid‐based tumor invasion assay. Details are reported under ‘Materials and methods’. Cultures were observed under light microscope and images were taken at 6 days after seeding. The experiment was repeated three times, while the figure reports the results of two replicates. On the right and on the left, images were obtained at different magnification. On the center, the diameter of the colonies obtained was measured using the scale bar (50 µm) as reference. The results shown are the mean of 3 determinations obtained on three independent experiments and standard deviation is showed. Data were analyzed by Student's *t* test. *P < 0.05.

**Fig. S4.** Apoptosis analysis of cells expressing G9R‐p27 compared to wt‐p27. PC‐3 cells were transfected for 48 h with pcDNA3.0 empty vector, and pcDNA3.0 encoding p27, or G9R‐p27. Then, cells were treated for 18 h with 1 µm staurosporine. Cells were collected and processed with Alexa Fluor 488 Annexin V/Dead Cell Apoptosis Kit according to manufacturer's indications. The control of this experiment is made by cells transfected with empty vector and treated with staurosporine (STAUROSPORINE) as reported under ‘Materials and methods’. Cell apoptosis was detected by flow cytometry using a FACScalibur and calculated analyzing 50 000 events. Upper right (UR) quadrant includes apoptotic cells.

**Fig. S5.** Bidimensional analysis of transfected mutants of G9R‐p27. (A) 2D/WB analysis of cell extracts of PC‐3 cells transfected with pcDNA3.0 plasmids encoding S10A/G9R‐p27, S10A/T187A/G9R‐p27 [*T187A], S10A/T198V/G9R‐p27 [*T198V], S10A/T157A/G9R‐p27 [*T157A], and S10A/Y(74,88,89)F/G9R‐p27 [*Y(74,88,89)F] on the left, and plasmids encoding S10A/G9R‐p27, S7A/S10A/G9R‐p27 [*S7A], S183A/S10A/G9R‐p27 [*S183A], S83A/S10A/G9R‐p27 [*S83A] on the right. After blotting, the filters were analyzed by mAb anti‐p27. Signals 0 and 1correspond to unmodified and 1Pi‐protein, respectively. (B) HeLa cells were transfected with pcDNA3.0 plasmids encoding G9R‐p27, S10A/G9R‐p27 [*S10A], and S12A/G9R‐p27 [*S12A]. Cell extracts were prepared and analyzed by 2D/WB. After blotting, the filters were analyzed by mAb anti‐p27. Signals 0, 1, and 2 correspond to unmodified, 1Pi‐ and 2Pi‐protein, respectively. (C) On the left. 2D/WB analysis of cell extracts of K562 cells transfected with pcDNA3.0 plasmids encoding G9R‐p27 and S12A/G9R‐p27 [*S12A]. After blotting, the filters were analyzed by mAb anti‐p27. Signals 0, 1, and 2 correspond to unmodified, 1Pi‐ and 2Pi‐protein, respectively. On the right. 2D/WB analysis of cell extracts of SH‐SY5Y cells transfected with pcDNA3.0 plasmids encoding G9R‐p27. After blotting, the filters were analyzed by mAb anti‐p27. Signals 0, 1, and 2 correspond to unmodified, 1Pi‐ and 2Pi‐protein, respectively. (D) On the left. 2D/WB analysis of cell extracts of K562 cells transfected with pcDNA3.0 plasmids encoding p27 protein, and its derivatives S12A/p27 [*S12A], and S12D/p27 [*S12D]. On the right. The histograms report the intensity percentage of each signal (unmodified, 1Pi‐isoforms) relative to the total for p27 and its derivative mutant proteins. The intensity of the specific signals was evaluated using TotalLab CLIQS gel image analysis Software. The data shown are the results of three independent experiments. Bars represent standard deviation. Data were analyzed by Student's *t* test. **P < 0.01.

**Fig. S6.** Effect of CDK2 on the nuclear and cytosolic localization of G9R‐p27. (A) Different population of MEF cells were investigated for confirming the absence of CDK2 and CDK4 protein. Immortalized wild type MEFs, and MEFs lacking CDK4, or CDK2 or both CDK4 and CDK2 were cultured as in Materials and methods. Then, the nuclear and cytosol compartments were prepared and analyzed for CDK2 and CDK4 by WB and specific antibodies. The filters were also analyzed for HDAC and PKM2 content by specific antibodies in order to confirm equal loading and compartment separation. (B) Upper figure. pcDNA3.0 plasmid encoding G9R‐p27 was transfected in different MEF populations, namely MEF immortalized cells, CDK4^−/−^ MEFs, CDK2^−/−^ and CDK4^−/−^CDK2^−/−^ cells. After 24 h, nuclear and cellular compartments were prepared and analyzed by WB employing mAb anti‐p27. HDAC1 was investigated for evaluating loading amount and nuclear purity. Lower figure. Three experiments similar to that reported on the top were performed. The percentage of nuclear and cytosolic protein was evaluated by imagej software. On the basis of determined data, the showed histograms were constructed. Error bars represent the standard error of the mean of the experiments. (C) pcDNA3.0 plasmids encoding p27 and G9R‐p27 were transfected in parental and CDK2^−/^CDK4^−^ MEFs. After 24 h, nuclear and cytosol extracts were prepared and analyzed by 2D/WB with mAb anti‐p27. For the nuclear extracts, images at different film exposition times are reported. (D) S10/S12/T187A/G9R‐p27 protein was prepared from PC‐3 transfected cells. The partially purified protein was incubated with recombinant CDK2 for 40 min. The assay mixtures at time 0 and after 40 min were analyzed by 2D/WB.
