# Supplementary figures and images for: A cancer‐associated CDKN1B mutation induces p27 phosphorylation on a novel residue: a new mechanism for tumor suppressor loss‐of‐function
Source: Mol Oncol. 2021 Feb 6;15(4):915–41. doi: 10.1002/1878-0261.12881 (PMC8024736; doi:10.1002/1878-0261.12881)

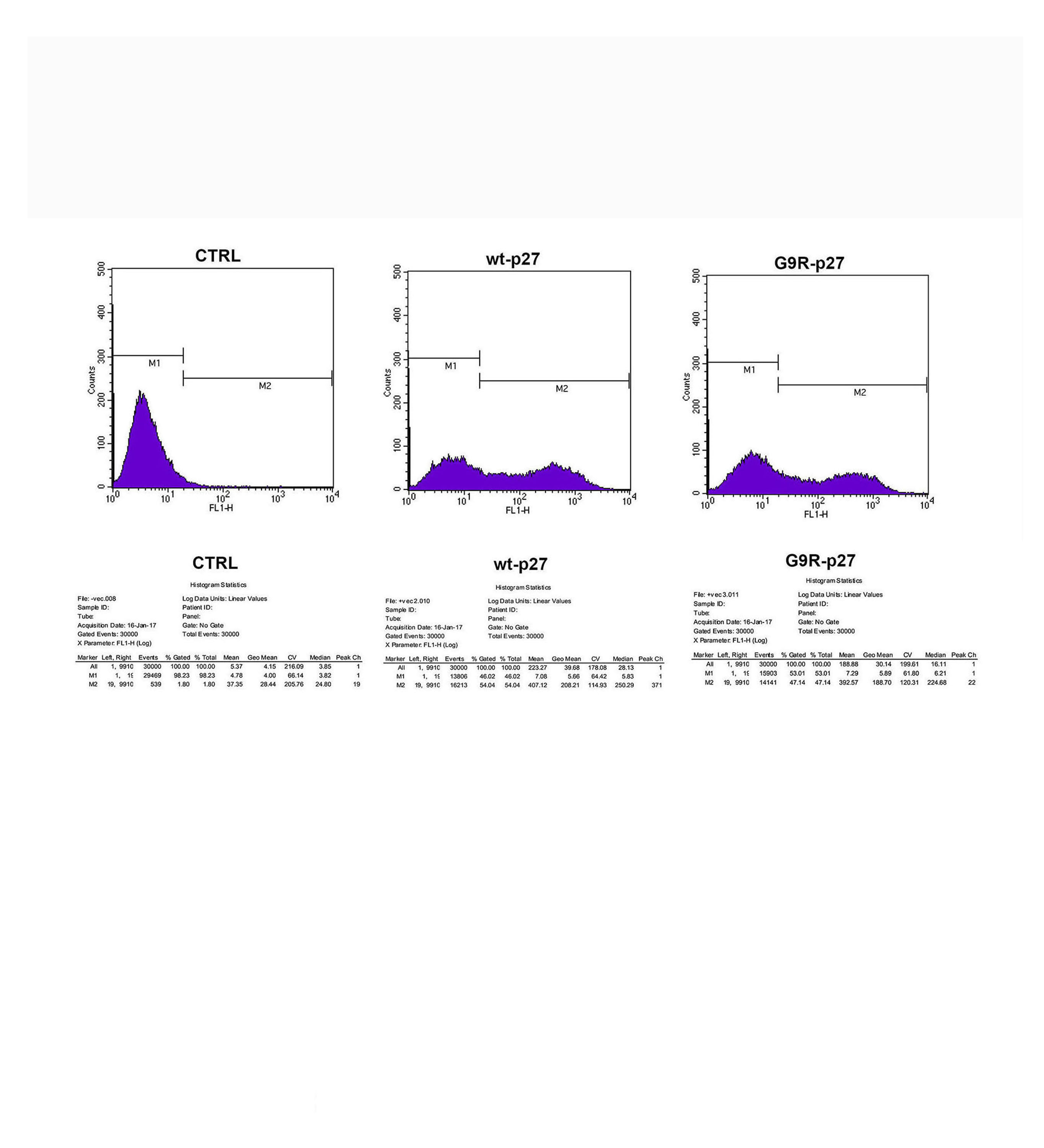

Supplement: Supplementary file 1 — Fig. S1. Transfection efficiency evaluation. HEK‐293 cells transfected for 48 h with empy‐vector (CTRL), wt‐p27 and G9R‐p27 were stained with anti‐p27 mAb and fluorescence‐tagged secondary antibodies and analyzed by flow cytometry using a FACScalibur. Calculations were done over 30 000 events. M2 includes wt‐ and mutated p27 expressing cells, corresponding at least to 50% of the whole cell populations. M1 comprises cells with a very low level of fluorescence corresponding to endogenous p27 staining. [file MOL2-15-915-s002.jpg]

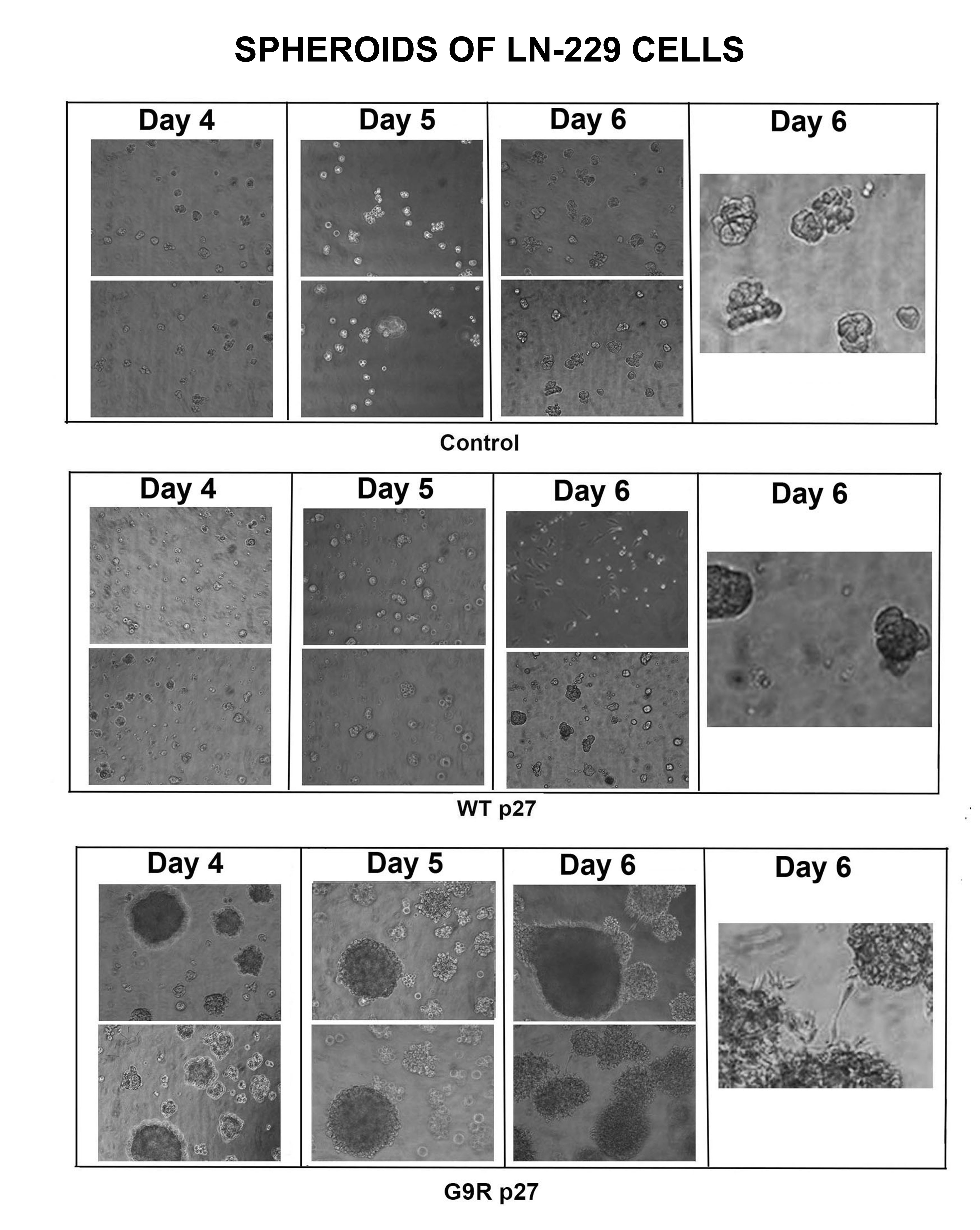

Supplement: Supplementary file 2 — Fig. S2. Spheroid formation ability of cells expressing wt‐p27 and G9R‐p27. LN‐229 glioblastoma cells transfected the day before with empty vector (CTRL) or plasmids encoding WT‐, and G9R‐p27 were seeded in matrigel for 3D spheroid‐based tumor invasion assay. Details are reported under ‘Materials and methods’. Cultures were observed under light microscope and images were taken at 4, 5 and 6 days after seeding. The experiment was repeated three times, while the figure reports the results of two replicates for each time point. On the right, images obtained after 6 days inclusion at higher magnification: G9R‐expressing cultures show the presence of cells that appear detaching from spheres (protruding cells). [file MOL2-15-915-s003.jpg]

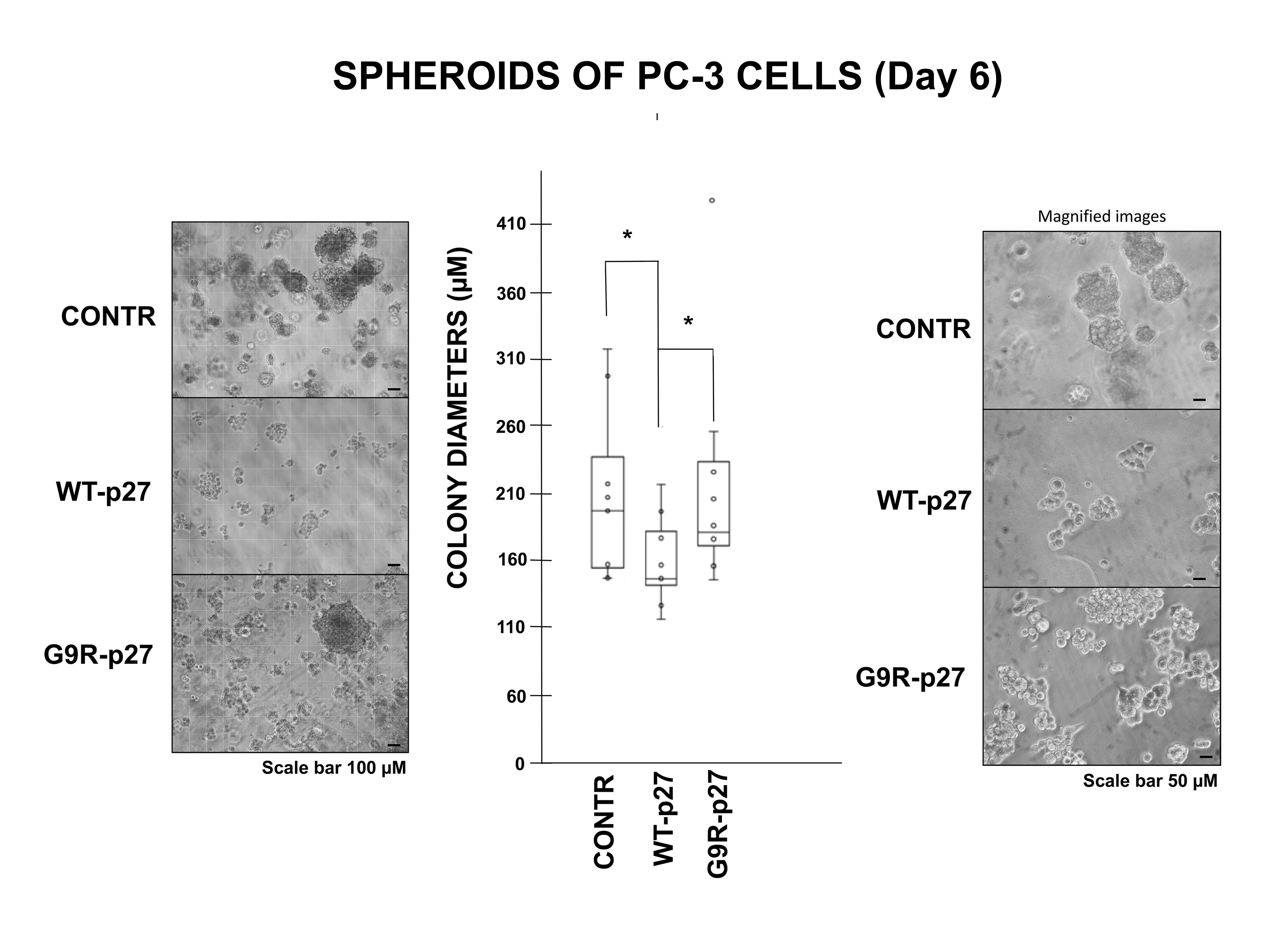

Supplement: Supplementary file 3 — Fig. S3. Spheroid formation ability of PC‐3 cells expressing wt‐p27 and G9R‐p27. PC‐3 cells transfected the day before with empty vector (CTRL) or plasmids encoding WT‐, and G9R‐p27 were seeded in matrigel for 3D spheroid‐based tumor invasion assay. Details are reported under ‘Materials and methods’. Cultures were observed under light microscope and images were taken at 6 days after seeding. The experiment was repeated three times, while the figure reports the results of two replicates. On the right and on the left, images were obtained at different magnification. On the center, the diameter of the colonies obtained was measured using the scale bar (50 µm) as reference. The results shown are the mean of 3 determinations obtained on three independent experiments and standard deviation is showed. Data were analyzed by Student's t test. *P < 0.05. [file MOL2-15-915-s005.jpg]

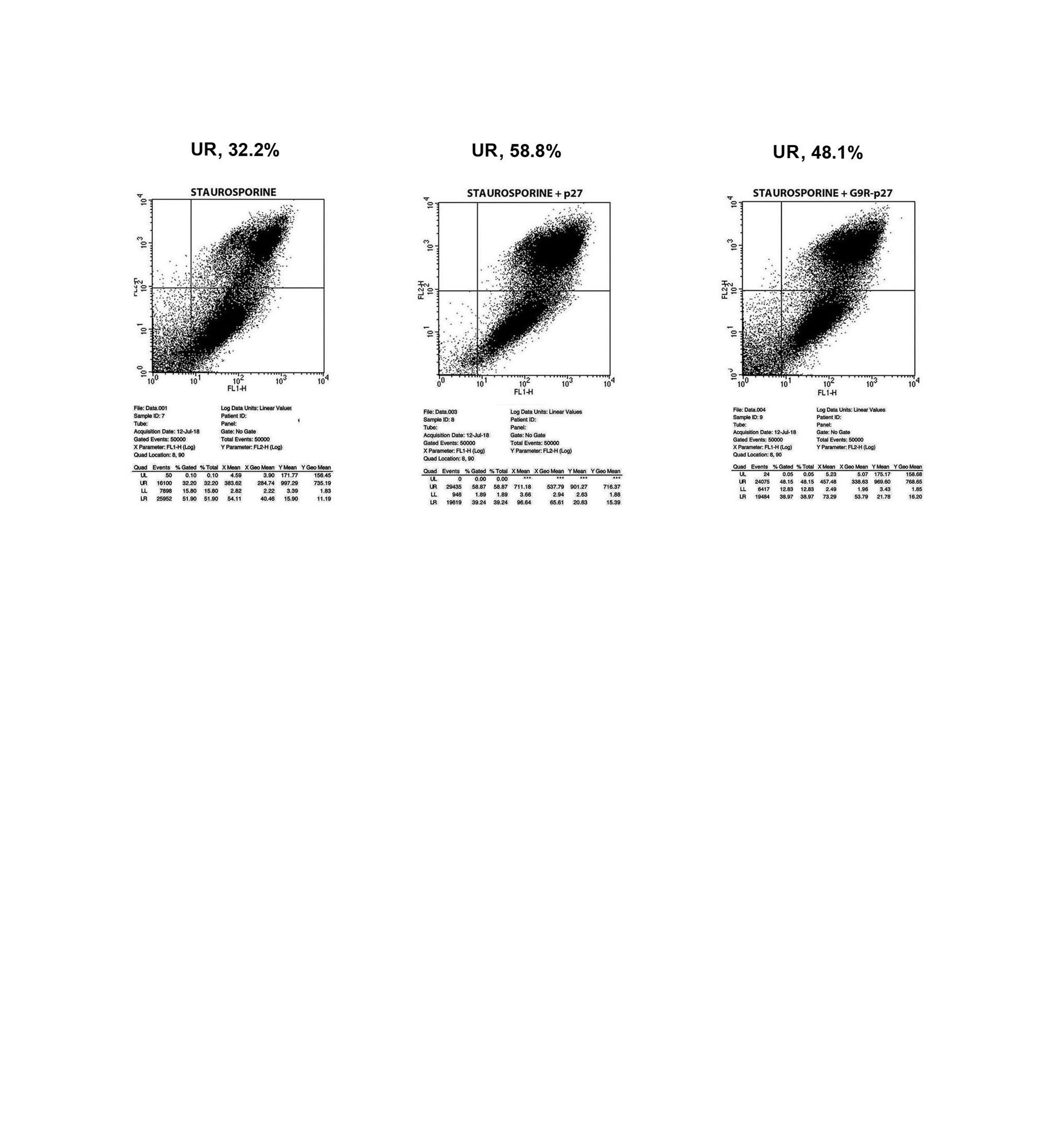

Supplement: Supplementary file 4 — Fig. S4. Apoptosis analysis of cells expressing G9R‐p27 compared to wt‐p27. PC‐3 cells were transfected for 48 h with pcDNA3.0 empty vector, and pcDNA3.0 encoding p27, or G9R‐p27. Then, cells were treated for 18 h with 1 µm staurosporine. Cells were collected and processed with Alexa Fluor 488 Annexin V/Dead Cell Apoptosis Kit according to manufacturer's indications. The control of this experiment is made by cells transfected with empty vector and treated with staurosporine (STAUROSPORINE) as reported under ‘Materials and methods’. Cell apoptosis was detected by flow cytometry using a FACScalibur and calculated analyzing 50 000 events. Upper right (UR) quadrant includes apoptotic cells. [file MOL2-15-915-s006.jpg]

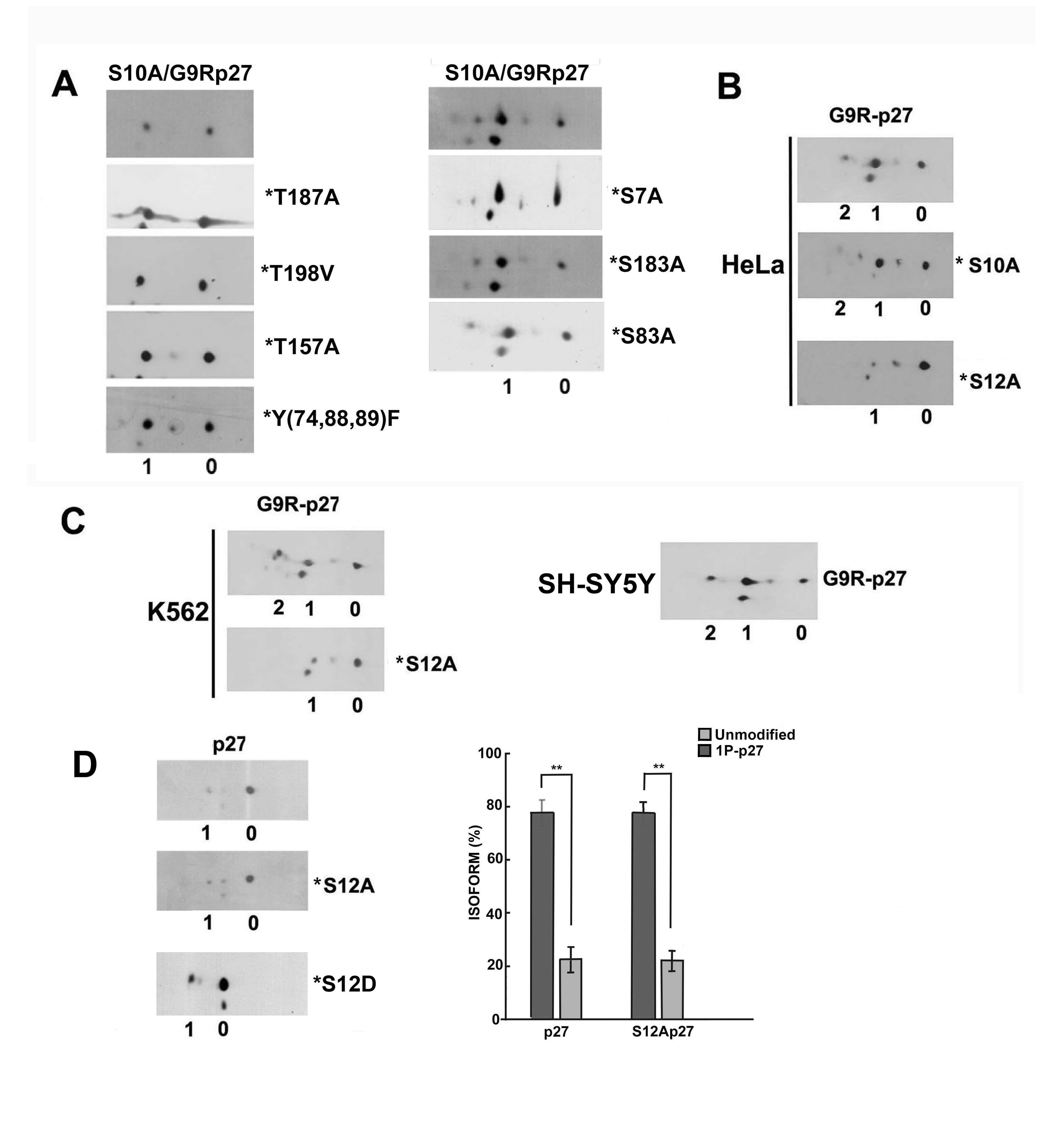

Supplement: Supplementary file 5 — Fig. S5. Bidimensional analysis of transfected mutants of G9R‐p27. (A) 2D/WB analysis of cell extracts of PC‐3 cells transfected with pcDNA3.0 plasmids encoding S10A/G9R‐p27, S10A/T187A/G9R‐p27 [*T187A], S10A/T198V/G9R‐p27 [*T198V], S10A/T157A/G9R‐p27 [*T157A], and S10A/Y(74,88,89)F/G9R‐p27 [*Y(74,88,89)F] on the left, and plasmids encoding S10A/G9R‐p27, S7A/S10A/G9R‐p27 [*S7A], S183A/S10A/G9R‐p27 [*S183A], S83A/S10A/G9R‐p27 [*S83A] on the right. After blotting, the filters were analyzed by mAb anti‐p27. Signals 0 and 1correspond to unmodified and 1Pi‐protein, respectively. (B) HeLa cells were transfected with pcDNA3.0 plasmids encoding G9R‐p27, S10A/G9R‐p27 [*S10A], and S12A/G9R‐p27 [*S12A]. Cell extracts were prepared and analyzed by 2D/WB. After blotting, the filters were analyzed by mAb anti‐p27. Signals 0, 1, and 2 correspond to unmodified, 1Pi‐ and 2Pi‐protein, respectively. (C) On the left. 2D/WB analysis of cell extracts of K562 cells transfected with pcDNA3.0 plasmids encoding G9R‐p27 and S12A/G9R‐p27 [*S12A]. After blotting, the filters were analyzed by mAb anti‐p27. Signals 0, 1, and 2 correspond to unmodified, 1Pi‐ and 2Pi‐protein, respectively. On the right. 2D/WB analysis of cell extracts of SH‐SY5Y cells transfected with pcDNA3.0 plasmids encoding G9R‐p27. After blotting, the filters were analyzed by mAb anti‐p27. Signals 0, 1, and 2 correspond to unmodified, 1Pi‐ and 2Pi‐protein, respectively. (D) On the left. 2D/WB analysis of cell extracts of K562 cells transfected with pcDNA3.0 plasmids encoding p27 protein, and its derivatives S12A/p27 [*S12A], and S12D/p27 [*S12D]. On the right. The histograms report the intensity percentage of each signal (unmodified, 1Pi‐isoforms) relative to the total for p27 and its derivative mutant proteins. The intensity of the specific signals was evaluated using TotalLab CLIQS gel image analysis Software. The data shown are the results of three independent experiments. Bars represent standard deviation. Data were analyz [file MOL2-15-915-s001.jpg]

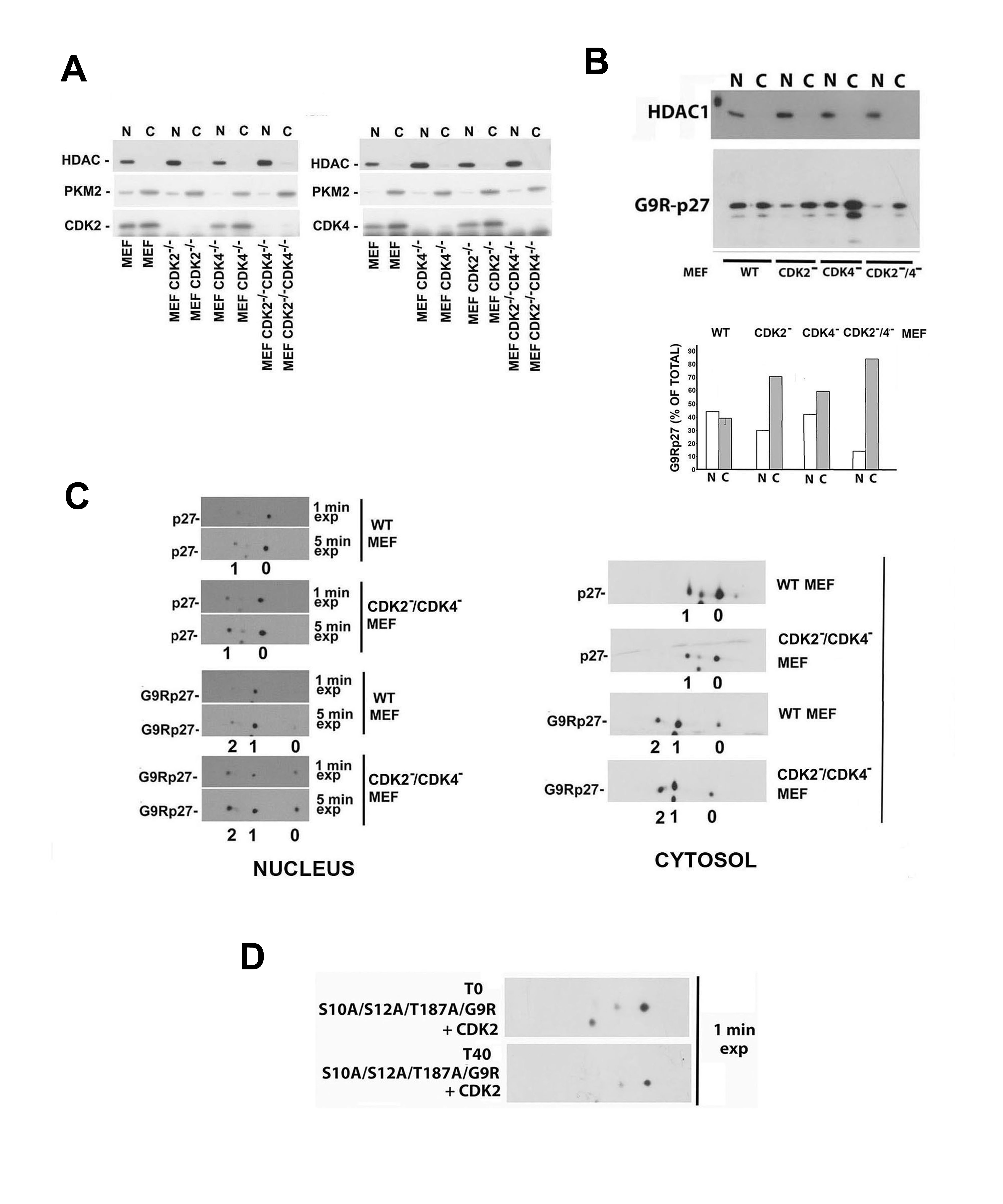

Supplement: Supplementary file 6 — Fig. S6. Effect of CDK2 on the nuclear and cytosolic localization of G9R‐p27. (A) Different population of MEF cells were investigated for confirming the absence of CDK2 and CDK4 protein. Immortalized wild type MEFs, and MEFs lacking CDK4, or CDK2 or both CDK4 and CDK2 were cultured as in Materials and methods. Then, the nuclear and cytosol compartments were prepared and analyzed for CDK2 and CDK4 by WB and specific antibodies. The filters were also analyzed for HDAC and PKM2 content by specific antibodies in order to confirm equal loading and compartment separation. (B) Upper figure. pcDNA3.0 plasmid encoding G9R‐p27 was transfected in different MEF populations, namely MEF immortalized cells, CDK4−/− MEFs, CDK2−/− and CDK4−/−CDK2−/− cells. After 24 h, nuclear and cellular compartments were prepared and analyzed by WB employing mAb anti‐p27. HDAC1 was investigated for evaluating loading amount and nuclear purity. Lower figure. Three experiments similar to that reported on the top were performed. The percentage of nuclear and cytosolic protein was evaluated by imagej software. On the basis of determined data, the showed histograms were constructed. Error bars represent the standard error of the mean of the experiments. (C) pcDNA3.0 plasmids encoding p27 and G9R‐p27 were transfected in parental and CDK2−/CDK4− MEFs. After 24 h, nuclear and cytosol extracts were prepared and analyzed by 2D/WB with mAb anti‐p27. For the nuclear extracts, images at different film exposition times are reported. (D) S10/S12/T187A/G9R‐p27 protein was prepared from PC‐3 transfected cells. The partially purified protein was incubated with recombinant CDK2 for 40 min. The assay mixtures at time 0 and after 40 min were analyzed by 2D/WB. [file MOL2-15-915-s004.jpg]
